# The landscape of tumor cell states and spatial organization in H3-K27M mutant diffuse midline glioma across age and location

**DOI:** 10.1038/s41588-022-01236-3

**Published:** 2022-12-05

**Authors:** Ilon Liu, Li Jiang, Erik R. Samuelsson, Sergio Marco Salas, Alexander Beck, Olivia A. Hack, Daeun Jeong, McKenzie L. Shaw, Bernhard Englinger, Jenna LaBelle, Hafsa M. Mire, Sibylle Madlener, Lisa Mayr, Michael A. Quezada, Maria Trissal, Eshini Panditharatna, Kati J. Ernst, Jayne Vogelzang, Taylor A. Gatesman, Matthew E. Halbert, Hana Palova, Petra Pokorna, Jaroslav Sterba, Ondrej Slaby, Rene Geyeregger, Aaron Diaz, Izac J. Findlay, Matthew D. Dun, Adam Resnick, Mario L. Suvà, David T. W. Jones, Sameer Agnihotri, Jessica Svedlund, Carl Koschmann, Christine Haberler, Thomas Czech, Irene Slavc, Jennifer A. Cotter, Keith L. Ligon, Sanda Alexandrescu, W. K. Alfred Yung, Isabel Arrillaga-Romany, Johannes Gojo, Michelle Monje, Mats Nilsson, Mariella G. Filbin

**Affiliations:** 1 Department of Pediatric Oncology, Dana-Farber Boston Children’s Cancer and Blood Disorders Center, Boston, MA USA; 2 Broad Institute of MIT and Harvard, Cambridge, MA USA; 3 Science for Life Laboratory, Department of Biochemistry and Biophysics, Stockholm University, Stockholm, Sweden; 4 Center for Neuropathology, Ludwig-Maximilians-University, Munich, Germany; 5 Department of Urology, Comprehensive Cancer Center, Medical University of Vienna, Vienna, Austria; 6 Department of Pediatrics and Adolescent Medicine, Comprehensive Center for Pediatrics and Comprehensive Cancer Center, Medical University of Vienna, Vienna, Austria; 7 Department of Neurology and Neurological Sciences, Stanford University School of Medicine, Stanford, CA USA; 8 Hopp Children’s Cancer Center Heidelberg (KiTZ), Division of Pediatric Glioma Research, German Cancer Research Center (DKFZ), Heidelberg, Germany; 9 Department of Oncologic Pathology, Dana-Farber Cancer Institute, Boston, MA USA; 10 Department of Neurological Surgery, University of Pittsburgh School of Medicine, Pittsburgh, PA USA; 11 John G. Rangos Sr. Research Center, Children’s Hospital of Pittsburgh, Pittsburgh, PA USA; 12 Central European Institute of Technology, Masaryk University, Brno, Czech Republic; 13 Pediatric Oncology Department, University Hospital Brno, Faculty of Medicine, Masaryk University, ICRC, Brno, Czech Republic; 14 Department of Biology, Faculty of Medicine, Masaryk University, Brno, Czech Republic; 15 Department of Clinical Cell Biology and FACS Core Unit, St. Anna Children’s Cancer Research Institute (CCRI), Vienna, Austria; 16 Department of Neurological Surgery, University of California San Francisco, San Francisco, CA USA; 17 Cancer Signalling Research Group, School of Biomedical Sciences and Pharmacy, College of Health, Medicine and Wellbeing, University of Newcastle, Callaghan, New South Wales Australia; 18 Precision Medicine Program, Hunter Medical Research Institute, New Lambton Heights, New South Wales Australia; 19 Center for Data Driven Discovery in Biomedicine, Children’s Hospital of Philadelphia, Philadelphia, PA USA; 20 Department of Pathology, Center for Cancer Research, Massachusetts General Hospital, Boston, MA USA; 21 Division of Pediatric Hematology/Oncology, Department of Pediatrics, Michigan Medicine, Ann Arbor, MI USA; 22 Division of Neuropathology and Neurochemistry, Department of Neurology, Medical University of Vienna, Vienna, Austria; 23 Department of Neurosurgery, Medical University of Vienna, Vienna, Austria; 24 Department of Pathology and Laboratory Medicine, Children’s Hospital Los Angeles, Keck School of Medicine of University of Southern California, Los Angeles, CA USA; 25 Department of Pathology, Brigham and Women’s Hospital, Boston, MA USA; 26 Department of Pathology, Boston Children’s Hospital, Boston, MA USA; 27 Department of Neuro-Oncology, Brain Tumor Center, The University of Texas MD Anderson Cancer Center, Houston, TX USA; 28 Massachusetts General Hospital, Cancer Center, Boston, MA USA; 29 Howard Hughes Medical Institute, Stanford, CA USA

**Keywords:** CNS cancer, Transcriptomics, Ageing, Cancer microenvironment

## Abstract

Histone 3 lysine27-to-methionine (H3-K27M) mutations most frequently occur in diffuse midline gliomas (DMGs) of the childhood pons but are also increasingly recognized in adults. Their potential heterogeneity at different ages and midline locations is vastly understudied. Here, through dissecting the single-cell transcriptomic, epigenomic and spatial architectures of a comprehensive cohort of patient H3-K27M DMGs, we delineate how age and anatomical location shape glioma cell-intrinsic and -extrinsic features in light of the shared driver mutation. We show that stem-like oligodendroglial precursor-like cells, present across all clinico-anatomical groups, display varying levels of maturation dependent on location. We reveal a previously underappreciated relationship between mesenchymal cancer cell states and age, linked to age-dependent differences in the immune microenvironment. Further, we resolve the spatial organization of H3-K27M DMG cell populations and identify a mitotic oligodendroglial-lineage niche. Collectively, our study provides a powerful framework for rational modeling and therapeutic interventions.

## Abstract (web summary)

Single-cell RNA-seq and ATAC-seq, combined with spatial transcriptomics, identify age- and location-related cellular dynamics of diffuse midline gliomas, such as variable oligodendrocyte precursor-like tumor stem cell populations and increased mesenchymal states with age.

## Main

Diffuse midline gliomas (DMG) driven by a lysine27-to-methionine (K27M) mutation in histone 3 (H3) are among the most lethal brain tumors^1–5^. Primarily identified in younger children (<10 years), the same oncohistone mutation is also recurrently observed in midline gliomas in adults^6–8^. In children, the spatiotemporal pattern of H3-K27M DMG incidence, peaking at 6–9 years of age in the brainstem pontine region, has shaped the hypothesis that the cell-intrinsic and -extrinsic context in which the K27M mutation occurs and elicits oncogenic transformation is developmental stage specific^9^. Indeed, previous studies have hinted at precursor cells in the pons^10^ and an early neurodevelopmental window^11^ as spatiotemporal correlates in K27M mutation-mediated gliomagenesis. Cell-intrinsically, the K27M mutation leads to broad epigenetic dysregulation and thus transformation of a developmentally restricted cell to a tumorigenic stem-like state^12–18^. The resulting active chromatin landscape reflects an early oligodendroglial lineage^19,20^. Single-cell RNA-sequencing (scRNA-seq) of pediatric, predominantly pontine H3-K27M tumors, further demonstrated that most glioma cells are stalled in a cancer stem cell-like oligodendrocyte precursor cell (OPC)-like state that is capable of self-renewal and tumor initiation^21,22^. In contrast, more differentiated noncycling glia-like cells were shown to have lost their tumorigenic capacity^21^. Together, this indicates OPC-like cells to be at the core of K27M mutation-mediated tumorigenesis, and hence, may present a strategic therapeutic target in pediatric pontine H3-K27M DMGs.

However, it remains incompletely understood whether H3-K27M DMGs of different midline locations—such as thalamus, pons or spinal cord—as well as different age groups and different morphological features at presentation, have similar cellular compositions. In particular, the more recently recognized group of adolescent (10–19 years) and adult (≥20 years) H3-K27M DMGs remains understudied. In addition to cell-intrinsic modes of dysregulation, mounting evidence indicates that microenvironmental factors critically contribute to glioma growth^23–28^, and it has been suggested that the developing brain provides a permissive environment that can be exploited for pediatric brain tumor growth^29,30^. However, the interplay between age- and region-specific tissue environments and the varying clinico-anatomical characteristics of H3-K27M DMGs, and its contribution to tumor pathology remain unexplored.

To address these questions, we have utilized single-cell multi-omics and spatial transcriptomic approaches to profile an extended cohort of H3-K27M DMGs encompassing a broad range of age groups and anatomical locations. We thereby identify how age- and location-dependent contexts underlie cell-intrinsic and -extrinsic features that together determine variation in glioma spatial and cellular architecture in light of the common K27M mutation.

## Results

### Cohort of H3-K27M DMGs across age groups and locations

**Fig. 1:**
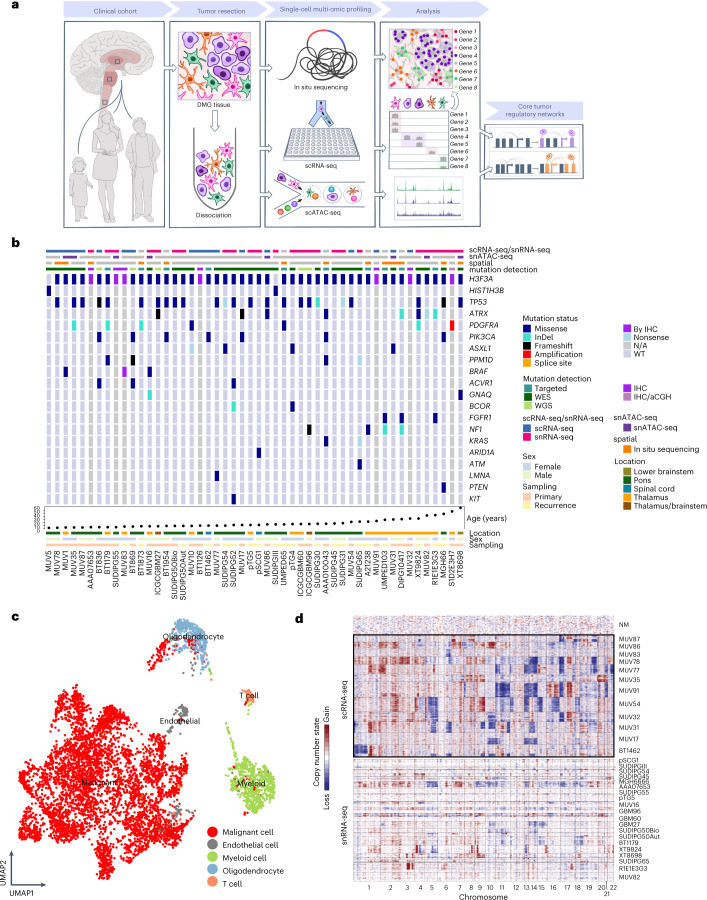
H3-K27M DMG cohort profiled by single-cell multi-omics. **a**, Schematic of the workflow. **b**, Clinico-molecular cohort characteristics. The upper legend bars depict the single-cell profiling method by scRNA-seq (*n* = 18)/snRNA-seq (*n* = 25), snATAC-seq (*n* = 8) and/or single-cell in situ sequencing (*n* = 14). The lower row specifies the method of genetic characterization. Most frequently detected and previously reported co-mutations are shown in the middle for 43 of 50 tumors profiled by whole or targeted exome sequencing. Clinico-anatomical characteristics are shown by the bottom legend bars. **c**, UMAP of all cells profiled by scRNA-seq/snRNA-seq. The color legend highlights malignant, types of nonmalignant cells detected based on clustering, copy number profiles and expression of canonical marker genes. For this visualization, scRNA-seq/snRNA-seq data were integrated by the Harmony algorithm, while downstream analyses were performed separately on scRNA-seq and snRNA-seq data to control for technical biases. **d**, Copy number alteration (CNA) profiles inferred from scRNA-seq/snRNA-seq data. Cells are ordered by their original tumors as rows and are clustered by their pattern of CNAs across chromosomal locations (columns). Representative fresh spike-in nonmalignant cells lacking CNAs are shown on top.

We conducted multi-omic profiling of 50 H3-K27M mutant patient tumors, selected only by criteria of the oncohistone mutation, spanning pontine (*n* = 27), thalamic (*n* = 20), lower brainstem (*n* = 1) and spinal (*n* = 2) locations (Fig. 1a,b and Supplementary Table 1). The median age was 12 (2.5–68) years, encompassing 36 pediatric (18 early childhood (0–9 years), 18 adolescent (10–19 years)) and 14 adult (20–68 years) tumors. Samples were obtained pre-treatment (*n* = 30) and post-treatment (*n* = 20) from 29 female and 21 male patients. We performed deep full-length Smart-seq2 fresh single-cell (*n* = 18) or frozen single-nucleus (*n* = 25) RNA-sequencing (scRNA-seq/snRNA-seq) of 43 tumors (Fig. 1a–c). We additionally analyzed the open chromatin profiles of eight tumors utilizing the single-cell/single-nucleus assay for transposase-accessible chromatin using sequencing (scATAC-seq/snATAC-seq), as well as the single-cell spatial transcriptomic architecture of 14 tumors by in situ sequencing (Fig. 1a,b).

To identify other mutations, we performed whole or targeted exome sequencing in 43 of 50 tumors (Fig. 1b). Recurrent mutations in *TP53, PDGFRA* and *PIK3CA* were broadly observed across all clinico-anatomical groups stratified by age and location, while alterations in *HIST1H3B* and *BRAF* were only rarely detected in childhood tumors, which is in line with previous reports of H3-K27M DMGs^1,5,8,31,32^.

Overall, our cohort covers a representative clinico-molecular range of H3-K27M DMGs. Interestingly, we did not detect significant differences in co-mutational profiles between different groups, and next set out to investigate non-genetic features and heterogeneity of H3-K27M DMGs across different spatiotemporal contexts.

### H3-K27M DMG cell composition across age and location

**Fig. 2:**
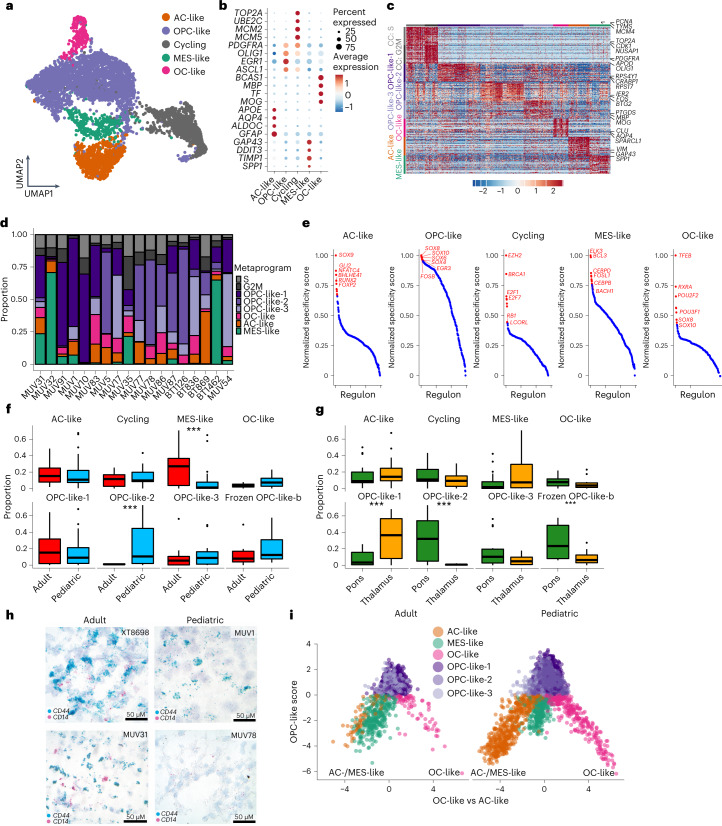
Intratumoral transcriptional heterogeneity of H3-K27M DMGs. **a**, UMAP of all fresh tumor cells, highlighting identified clusters. **b**, Marker genes (*y* axis) of identified fresh tumor cell clusters, grouped and annotated on the *x* axis. Dot sizes represent the percentage of cells expressing the gene in the given cluster, and the color scale shows scaled average relative expression. **c**, Heatmap representing the relative expression (color bar) of the top 30 marker genes (rows) for the tumor metaprograms identified by NMF across all fresh tumor cells (columns). **d**, Proportions (*y* axis) of fresh tumor-derived NMF metaprograms (color legend) in tumor cells for each fresh sample (*x* axis). **e**, Cell type-specific TF regulatory networks (regulon, *x* axis) derived by SCENIC, plotted against their normalized specificity score (*y* axis). **f**, Boxplots representing relative frequencies of metaprograms in all fresh and frozen tumors in adult (*n* = 10) versus pediatric (*n* = 23) age groups. The median is marked by the thick line within the boxplot, the first and third quartiles by the upper and lower limits, and the 1.5 times interquartile range by the whiskers. Three asterisks denote credible statistical changes as assessed by a Bayesian scCODA model with FDR < 0.05 and without multiple test corrections. **g**, Boxplots representing relative frequencies of metaprograms in all fresh and frozen tumors grouped by pontine (*n* = 19) versus thalamic (*n* = 14) locations. The median is marked by the thick line within the boxplot, the first and third quartiles by the upper and lower limits, and the 1.5 times interquartile range by the whiskers. Three asterisks denote credible statistical changes as assessed by a Bayesian scCODA model with FDR < 0.05 and without multiple test corrections. **h**, RNA in situ hybridization for MES-like (*CD44*) and macrophage (*CD14*) markers in two adult and two pediatric H3-K27M DMGs. Two to three slides were stained for each sample with 10–15 fields of view taken per slide. **i**, Two-dimensional representations of the OC-like versus AC-like (*x* axis) and OPC-like (*y* axis) scores for adult and pediatric H3-K27M DMGs, respectively.

**Extended Data Fig. 1:**
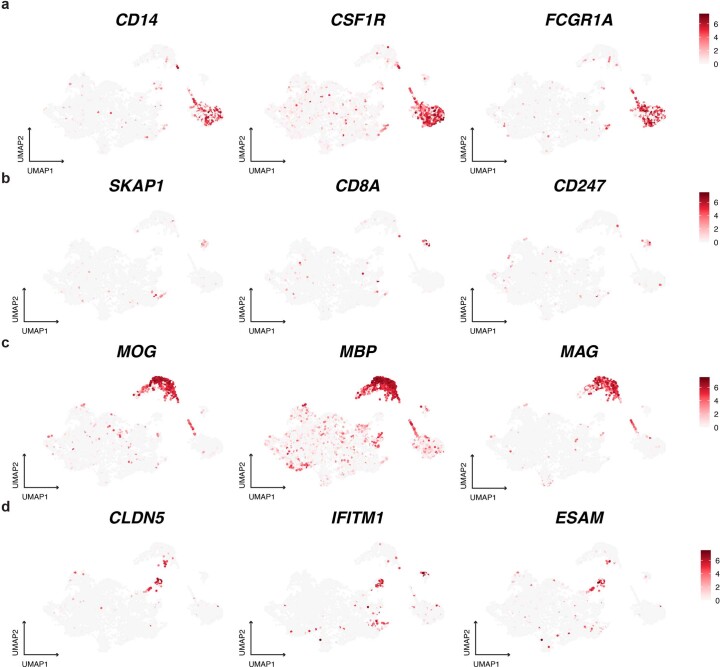
Non-malignant cell populations. UMAP projections highlighting non-malignant cell clusters by expression of canonical markers of (a) Tumor-associated myeloid cells. (b) T cells. (c) Oligodendrocytes. (d) Endothelial cells.

**Extended Data Fig. 2:**
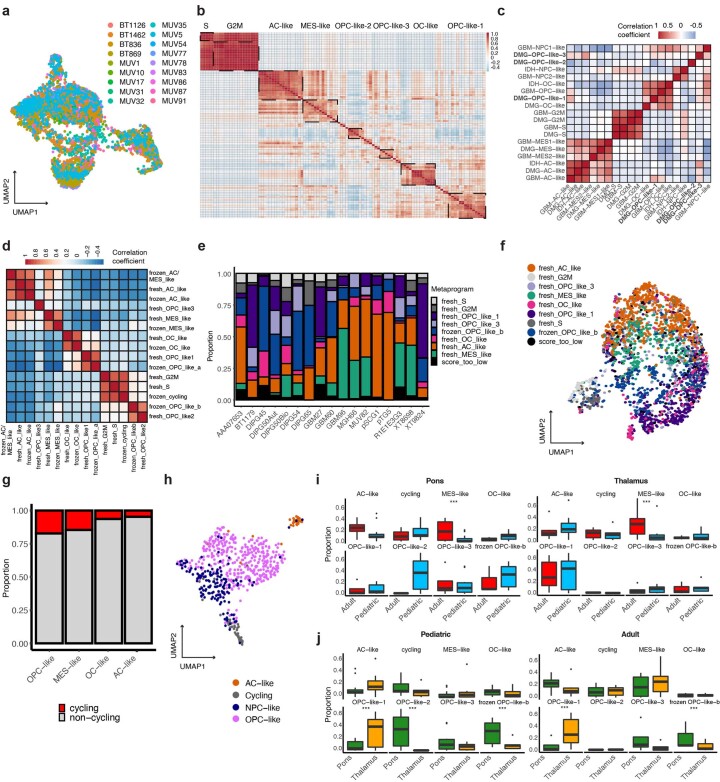
Intratumoral transcriptional heterogeneity of H3-K27M DMGs. (a) UMAP of fresh tumor cells, highlighting original samples (color legend) after batch effect correction. (b) Pairwise Pearson correlations (color scale) between NMF factors derived from each fresh tumor sample (x-axis). Highly correlated NMF factors were combined as metaprograms. (c) Pairwise Pearson correlations (color scale) between metaprograms derived from fresh H3-K27M DMGs, GBM^33^, IDH-mutant glioma^35^. (d) Pairwise Pearson correlations (color scale) between metaprograms independently derived from fresh and frozen tumors. (e) Proportions (y-axis) of projected fresh tumor derived metaprograms (color legend), that were highly correlated to respective frozen metaprograms, and of fresh OPC-like-3, across frozen tumor nuclei (x-axis). Instead of fresh OPC-like-2, correlated frozen OPC-like-b was scored to minimize technical ariefacts (see methods). Nuclei with scores <0.2 are denoted as ‘score too low’. (f) UMAP of frozen tumor nuclei after batch effect correction, with color legend depicting annotation based on single-cell scores of all fresh metaprograms and frozen OPC-like-b (see methods). (g) Proportion of all cells/nuclei assigned as cycling vs. non-cycling (color legend) across metaprograms. (h) UMAP of location matched IDH-mutant midline tumors, highlighting independently derived metaprograms. (i) Boxplots depicting metaprogram proportions in all tumors compared by adult vs. pediatric age groups, controlled for pontine (left) or thalamic (right) locations (Thalamic: adult (N = 6), pediatric (N = 8); Pontine: adult (N = 4), pediatric (N = 15)). (j) Boxplots depicting metaprogram proportions in all tumors compared by pontine and thalamic locations, controlled for pediatric (left) or adult (right) age groups (Adult: thalamic (N = 6), pontine (N = 4); Pediatric: thalamic (N = 8), pontine (N = 15)). In (i) and (j) The median is marked by the thick line within the boxplot, the first and third quartiles by the upper and lower limits, and the 1.5x interquartile range by the whiskers. *** denotes credible statistical changes as assessed by a Bayesian scCODA model, with FDR < 0.05, without multiple test correction.

We aimed at delineating and comparing transcriptional heterogeneity within our cohort stratified by age and location (Fig. 1c,d and Extended Data Fig. 1a–d). Complementary approaches assessing inter- and intratumoral heterogeneity concordantly identified tumor cells differentially expressing actively cycling, OPC-like, ‘astrocyte-likeʼ (AC-like), ‘oligodendrocyte-likeʼ (OC-like) and ‘mesenchymal-likeʼ (MES-like) signatures (Fig. 2a–d, Extended Data Fig. 2a–g and Supplementary Table 2). OPC-like cells were further resolved into three subpopulations (OPC-like-1, OPC-like-2 and OPC-like-3) (Fig. 2c,d and Extended Data Fig. 2b,c). Interestingly, the MES-like signature, which has been described in glioblastoma (GBM)^33,34^, has not been identified in H3-K27M DMGs before, hinting at unique properties uncovered from previously understudied clinico-anatomical groups within our extended cohort.

OPC-like cells were ubiquitously present in all tumors independent of age or location (Fig. 2a,d and Extended Data Fig. 2e,f). Interestingly, even in this expanded cohort, we did not detect any neuronal lineage tumor cells, placing this in contrast to all other high-grade glioma types and isocitrate dehydrogenase (IDH)-mutant glioma^33,35–37^. To investigate whether this may be a phenomenon specific to the midline location, we single-cell profiled two location- and age-matched IDH-mutant midline gliomas (Supplementary Table 1), revealing that neuronal lineage programs are present within rare midline IDH-mutant tumors (Extended Data Fig. 2h). Hence, this comparison of primary gliomas of the same location and age groups, but different genotypes, supports a direct cell-intrinsic effect of the K27M mutation to skew tumor cells toward a glial/OPC-like instead of a neuron-like identity.

We next reconstructed networks of active transcription factors (TFs) and their downstream gene targets (gene regulatory networks (GRNs)) (Fig. 2e and Supplementary Table 3) by single-cell regulatory network inference and analysis (SCENIC)^38^. We indeed found key GRNs known from normal glial specification (for example, SOX10 in OPC-like cells, TFEB in OC-like cells, SOX9 in AC-like cells) to be likewise active in respective H3-K27M DMG tumor cell counterparts, highlighting parallels between normal developmental and glioma cell fate determination. Moreover, we identify GRNs (for example, GLI2 and NFATC4) that have not yet been implicated in normal development and may hence present glioma-specific regulatory aberrations.

We next compared cellular compositions across tumor locations and age groups (Fig. 2f,g and Extended Data Fig. 2i,j). Interestingly, the MES-like metaprogram was substantially enriched in adult tumors (Fig. 2f), which persisted when we controlled for location as a potential confounding factor (Extended Data Fig. 2i). This was validated by RNA in situ hybridization (Fig. 2h). Except for one NF1*-*mutated pediatric tumor (Fig. 1b), which was associated with a stronger MES-like signature as previously reported^33,39^, we did not detect any additional recurring genetic mutations in coding gene regions in tumors enriched for MES-like cells, suggesting either non-coding mutations and/or non-genetic determinants may underlie the observed age-specificity. As such, this age-related difference points toward the emerging role of the tumor microenvironment in shaping the MES-like signature, as has been illustrated in recent studies^27,28,40^.

Together, we demonstrate that H3-K27M DMGs are biased toward an OPC-like cell identity independent of age or midline location, which suggests cell-intrinsic effects of the K27M oncohistone mutation itself rather than environmental determinants to underlie this cellular state. Contrastingly, an association with age is observed for the MES-like signature (Fig. 2i), potentially linking this cellular state to cell-extrinsic/environmental drivers.

### Location specificity of OPC-like subpopulations

**Fig. 3:**
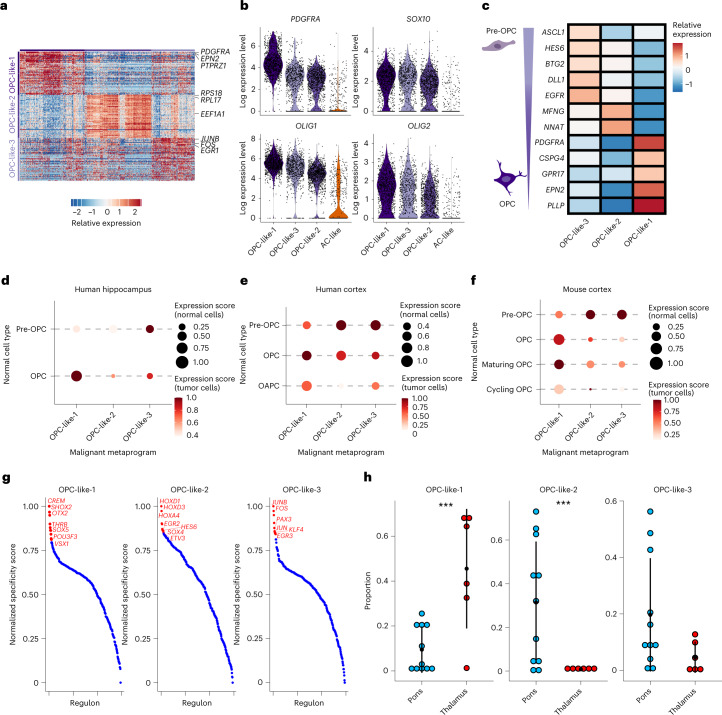
Region-specific states of OPC-like cells. **a**, Heatmap representing the relative expression (color scale) of the top 30 marker genes (rows) for the different OPC metaprograms across all fresh tumor cells (columns). **b**, Violin plots depicting log normalized absolute expressions of canonical OPC marker genes in OPC-like-1, OPC-like-2 and OPC-like-3 subpopulations. Expressions in AC-like cells (orange) are shown for comparison. **c**, Heatmap representing the relative expression (color scale) of canonical pre-OPC and OPC marker genes (rows) in tumor OPC-like-3, OPC-like-2 and OPC-like-1 populations (columns). **d**, Projection of OPC-like-1, OPC-like-2 and OPC-like-3 populations (*x* axis) onto normal pre-OPC and OPC (*y* axis) from a scRNA-seq dataset of the human hippocampus^46^. Color scale presents expression scores of normal cell signatures in tumor cells, while dot sizes depict expression scores of tumor cell signatures in normal cells. **e**, Projection of OPC-like-1, OPC-like-2 and OPC-like-3 populations (*x* axis) onto normal pre-OPC, OPC and OAPC (HOPX^+^SPARCL1^+^ glial progenitor cell) (*y* axis) from a scRNA-seq dataset of the human developing cortex^41^. Color scale presents expression scores of normal cell signatures in tumor cells, while dot sizes depict expression scores of tumor cell signatures in normal cells. **f**, Projection of OPC-like-1, OPC-like-2 and OPC-like-3 populations (*x* axis) onto different normal OPCs of varying maturation stages (*y* axis) from a scRNA-seq dataset of the neonatal mouse cortex^43^. Color scale presents expression scores of normal cell signatures in tumor cells, while dot sizes depict expression scores of tumor cell signatures in normal cells. **g**, TF regulatory networks (regulon, *x* axis) derived by SCENIC for each tumor OPC-like subpopulation, plotted against their normalized specificity score (*y* axis). **h**, Dotplots representing the distribution (mean ±2 × s.e.m.) of the proportions of different OPC-like tumor states across all fresh tumors grouped by pontine (*n* = 11) and thalamic (*n* = 6) locations. Three asterisks denote credible statistical changes as assessed by a Bayesian scCODA model, with FDR < 0.05 and without multiple test corrections.

**Extended Data Fig. 3:**
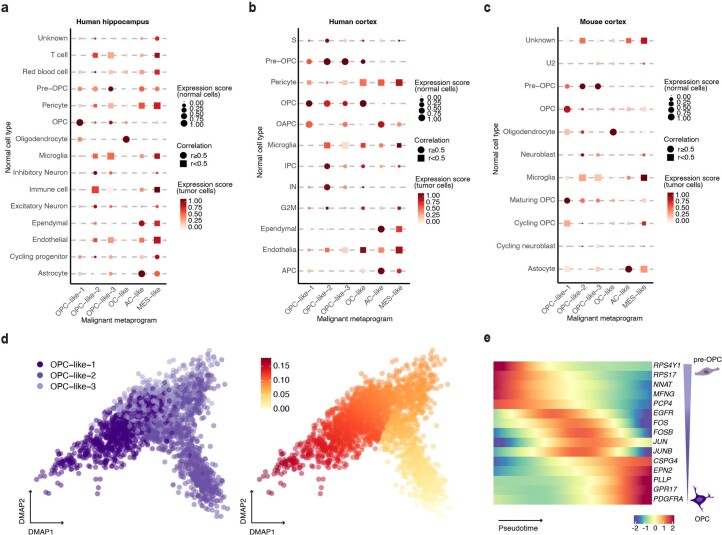
Region-specific states of OPC-like cells. (a) Projection of fresh tumor-derived metaprograms (x-axis) onto scRNA-seq derived normal cell types (y-axis) of the human hippocampus^43^. Color scale presents expression scores of normal cell signatures in tumor cells, while symbol sizes depict expression scores of tumor cell signatures in normal cells. Symbol shape denotes Pearson correlation of expressions, with circle denoting r > =0.5, and square denoting r < 0.5. (b) Projection of fresh tumor-derived metaprograms (x-axis) onto scRNA-seq derived normal cell types (y-axis) of the developing human cortex^40^. Color scale presents expression scores of normal cell signatures in tumor cells, while symbol sizes depict expression scores of tumor cell signatures in normal cells. Symbol shape denotes Pearson correlation of expressions, with circle denoting r > =0.5, and square denoting r < 0. (c) Projection of fresh tumor-derived metaprograms (x-axis) onto scRNA-seq derived normal cell types (y-axis) of the neonatal mouse cortex^42^. Color scale presents expression scores of normal cell signatures in tumor cells, while symbol sizes depict expression scores of tumor cell signatures in normal cells. Symbol shape denotes Pearson correlation of expressions, with circle denoting r > =0.5, and square denoting r < 0. (d) Diffusion map embedding of single OPC-like subpopulation transcriptomes (left) and pseudotime analysis by Slingshot where the color scale represents the relative pseudotime (right). (e) Heatmap representing Z-scored expression levels (color scale) of pre-OPC and OPC marker genes (rows) in tumor OPC-like subpopulations ordered along pseudotime (columns).

We next examined the three OPC-like subpopulations uniquely detected in our extended scRNA-seq dataset, termed OPC-like-1, OPC-like-2 and OPC-like-3 (Figs. 2c,d and 3a). While all OPC-like subpopulations were defined by high expression of canonical OPC markers (for example, *PDGFRA, SOX10* and *OLIG1/2*), these markers together with other known marker genes of committed OPCs (for example, *CSPG4, GPR17* and *EPN2*) were most highly expressed by OPC-like-1 cells (Fig. 3a–c)^41,42^. In contrast, OPC-like-2 and −3 cells depicted higher expression of marker genes linked to more immature oligodendrocyte precursors of the developing brain, also termed pre-OPCs—a state of oligodendroglial lineage differentiation between less differentiated neural stem cell and more differentiated OPC (for example, *ASCL1, HES6, BTG2, DLL1* and *EGFR*) (Fig. 3c)^41–43^. Additionally, OPC-like-2 cells highly expressed genes encoding ribosomal proteins (for example, *RPL17* and *RPS18*), and OPC-like-3 cells exhibited higher expression of immediate early response genes (for example, *JUNB* and *EGR1*) (Fig. 3a), which have been previously described as markers of different normal (pre-)OPC subpopulations^44,45^. When we projected these OPC-like subpopulations onto scRNA-seq atlases of the human telencephalon and mouse cortex^41,43,46^, the OPC-like-1 subpopulation indeed mapped to committed/maturing OPCs, whereas OPC-like-2 and OPC-like-3 cells were more similar to pre-OPCs (Fig. 3d–f and Extended Data Fig. 3a–c). Comparison with cell populations from other glioma types and trajectory analyses (Extended Data Fig. 2c; 3d,e) also pointed toward a more immature state of OPC-like-2 and OPC-like-3 cells, and stronger lineage commitment of OPC-like-1 cells.

Analysis of OPC-like subpopulation-specific GRNs using our scRNA-seq dataset identified TFs such as SHOX2 and OTX2 to be most specifically active in OPC-like-1 cells (Fig. 3g and Supplementary Table 3). GRNs specific to OPC-like-2 cells included Notch signaling regulator HES6 as well as multiple patterning TFs of the HOX family, and GRN characteristics of OPC-like-3 cells were linked to the AP-1 TF family (Fig. 3g). Of note, HOX patterning TFs have been demonstrated to be expressed in mice embryonal pre-OPCs while being downregulated in postnatal OPCs^44^. Moreover, immediate early response regulators have been implicated as specific to human pre-OPCs compared to committed OPCs^45^, further hinting at a more immature and pre-OPC-like state of DMG OPC-like-2 and OPC-like-3 cells.

We next compared proportions of these OPC-like subpopulations across our spatiotemporally stratified cohort and observed a remarkable enrichment of pre-OPC-like (OPC-like-2 and OPC-like-3) cells in pontine compared to thalamic tumors. Conversely, OPC-like-1 cells were enriched in thalamic tumors (Fig. 3h). These differences remained when stratifying for age groups as potential confounders (Extended Data Fig. 2j).

Therefore, we identify tumor location as a contextual determinant of OPC-like states, with immature pre-OPC-like progenitors enriched in pontine, and more committed OPC-like cells enriched in thalamic tumors.

### The open chromatin landscape of H3-K27M DMG cell populations

**Fig. 4:**
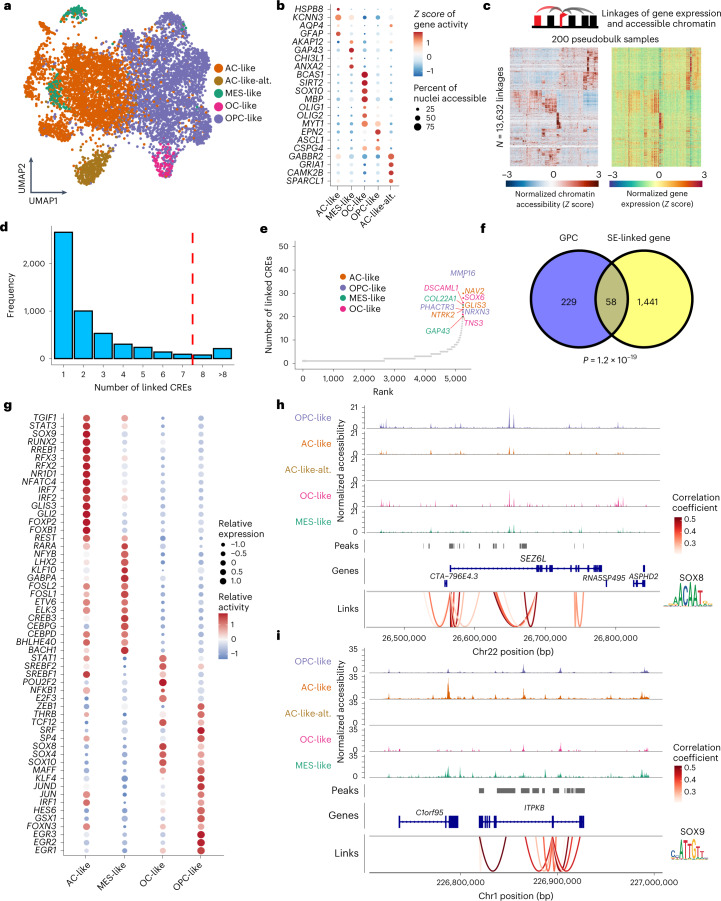
Characteristic chromatin profiles of H3-K27M DMG cell populations. **a**, UMAP of all snATAC-seq derived tumor nuclei after batch effect correction, highlighting de novo assigned clusters. **b**, Dotplot representation of top marker genes with differential gene activities (color scale) and proportion of nuclei accessible (dot size) within snATAC-seq derived cell states. **c**, Heatmap showing normalized chromatin accessibility and gene expressions of 13,632 substantially linked CRE-gene pairs (left rows, chromatin accessibility; right rows, linked gene expressions). Rows were clustered using hierarchical clustering. For visualization, 5,000 rows were randomly selected. **d**, Barplot representing distribution of numbers of linked CREs per gene. Red dashed line denotes the top 5% threshold of numbers of linked CREs that define GPC. **e**, Ranking of genes (*x* axis) by numbers of linked CREs (*y* axis) highlighting genes with top 20 linked CREs in color. Genes differentially expressed in a tumor cell state or identified as a cell state-specific TF regulon by SCENIC are colored according to the legend. **f**, Venn diagram representing overlap of GPCs with H3-K27M DMG super-enhancer associated genes, identified by Nagaraja et al.^19^. *P* value of a two-sided hypergeometric test is shown. **g**, Dotplot of integrative TF analysis representing the top cell state (columns)-specific TFs (rows). Average relative expression level assessed by scRNA-seq is depicted by dot size, and relative activity inferred by SCENIC analysis is presented by color scale. **h**,**i**, Integrative representation of gene loci of the **h**, OPC-like cell-specific *SEZ6L* gene, and **i**, AC-like cell-specific *ITPKB* gene. At the top, pseudobulk chromatin accessibility track plots are shown colored by cell type. In the middle row, bars depict the locus of putative CREs. In the bottom row, loops denote the correlation between chromatin accessibility of each peak and expression of its linked gene, representing putative CREs that are enriched for the OPC-like cell-specific SOX8 (**h**), AC-like cell-specific SOX9 (**i**), TF motifs, respectively.

**Extended Data Fig. 4:**
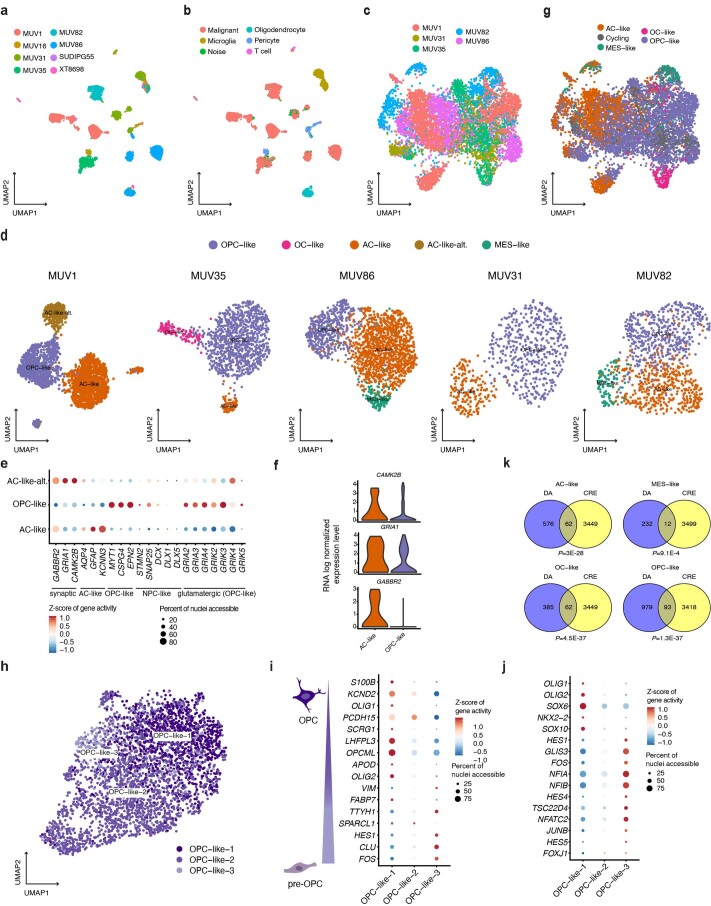
Characteristic chromatin profiles of H3-K27M DMG cell populations. (a) UMAP of all nuclei profiled by snATAC-seq from 8 samples (color legend). (b) UMAP of all nuclei profiled by snATAC-seq, highlighting assignments as malignant or different nonmalignant cell types. (c) UMAP of all snATAC-seq derived tumor nuclei, highlighting sample of origin after batch effect correction. (d) Sample level clustering analyses and de novo cell type annotations (color legends). (e) Dotplot representation of gene activities (color scale) and proportion of nuclei accessible (dot size) in snATAC-seq profiles of AC-like-alt., AC-like and OPC-like cells (y-axis) for canonical marker genes of AC-like, OPC-like, NPC-like (as identified in Neftel et al., 2019^33^), and glutamatergic (as described to be enriched in OPC-like cells by Venkatesh et al., 2019^25^) tumor cells. (f) ScRNA-seq derived log transformed expression levels of synapse-associated genes differentially accessible in AC-like-alt. cells. (g) Cell state annotations of all snATAC-seq tumor nuclei based on scRNA-seq data following canonical correlation (CCA) and label transfer analyses. (h) UMAP of chromatin accessibility profiles of all OPC-like subpopulations (color legend). (i) & (j) Dotplot representation of gene activities (color scale) and proportion of nuclei accessible (dot size) in snATAC-seq profiles of different tumor OPC-like subpopulations (x-axis) for top differentially accessible marker genes (i) and TFs (j) derived from studies of normal pre-OPCs and OPCs^45^. (k) Venn diagrams depicting the intersection of differentially accessible chromatin sites with CREs that are linked to GPCs for each cell type. p-values calculated from a two-sided hypergeometric test are shown.

To resolve how H3-K27M DMG cellular heterogeneity is governed at the chromatin level, we probed single-nucleus accessible chromatin profiles by snATAC-seq of eight tumors complementing their single-cell transcriptomes. De novo annotation of malignant cell clusters proved largely concordant with scRNA-seq-derived cell populations and included an additional group of AC-like (AC-like-alternative) cells with increased gene activity scores for synaptic marker genes (for example, *GABBR2, GRIA1* and *CAMK2B*) (Fig. 4a,b, Extended Data Fig. 4a–f and Supplementary Table 4; Supplementary Note). Cross-modality integration with scRNA-seq data further demonstrated overall congruence between chromatin- and transcriptome-defined cell states (Extended Data Fig. 4g). Notably, this also revealed distinct clusters of OPC-like-1, OPC-like-2 and OPC-like-3 cells in snATAC-seq space (Extended Data Fig. 4h). Concordant with our scRNA-seq findings, OPC-like-2 and OPC-like-3 cells also exhibited similarities with pre-OPCs at open chromatin level, whereas OPC-like-1 cells depicted higher chromatin accessibility for genes also described in healthy committed OPCs^45^ (Extended Data Fig. 4h–j). Thus, our finding of different OPC-like subpopulations is represented at both transcriptome and accessible chromatin levels.

As snATAC-seq resolves gene-distal and intragenic accessible chromatin regions containing potential *cis*-regulatory DNA elements (CREs) that underlie gene expression, we next inferred putative CREs integrating snATAC-seq and scRNA-seq modalities. By correlating snATAC-seq-derived accessible chromatin regions/peaks to scRNA-seq measured expression levels of their nearest associated gene (Fig. 4c)^47,48^, we identified 13,632 potential peak-gene links of CREs and their target genes (Supplementary Table 4 and Extended Data Fig. 4k). Among these, 287 genes exhibited more than eight (top 5%) linked CREs, denoting high regulatory locus complexity that has been described as ‘predictiveʼ chromatin and thereby a determinant of key lineage marker genes (Fig. 4d,e)^47,48^. We identified a higher number of genes linked with predictive chromatin (termed ‘GPCsʼ) specific to OPC/OC-like as compared to AC-like/MES-like cells, indicating highly cooperative regulation of the oligodendroglial lineage pervasively underlying H3-K27M DMGs (Fig. 4e; Methods). Because large groups of CREs are related to the concept of ‘super-enhancersʼ^47,48^, we overlayed our candidate GPCs with H3-K27ac ChIP-seq derived super-enhancer profiles of H3-K27M primary tumors^19^. This demonstrated a significant overlap of GPCs with H3-K27M DMG super-enhancer regulated genes (Fig. 4f), and further points toward a key role of these multimodally derived marker genes in orchestrating H3-K27M tumor cell identities.

We next sought to reconstruct and refine interdependent circuits of gene regulation by integrating expressions and activities of TFs inferred from scRNA-seq and enrichment of TF binding motifs in CREs derived from snATAC-seq (Methods). We identified 65 putative cell state-specific TFs that our analysis indicated to be (1) expressed at sufficient levels, (2) binding to characteristic motifs substantially enriched in CREs and (3) altering expressions of downstream target genes in a cell-type-specific manner (Fig. 4g). Moreover, we examined which TFs potentially regulate GPCs, focusing on TFs predicted to regulate expressions of GPCs and having binding sites detected within GPC-linked CREs. For example, the OPC-like marker gene *SEZ6L* is differentially expressed and accessible in OPC-like cells, and is linked to 16 CREs containing TF binding sites of SOX8, which is again predicted to be differentially active in OPC-like cells (Fig. 4h). We describe the same interdependencies between gene expression, chromatin accessibility and enrichment of cell state-specific TFs in CREs for GPCs of all tumor cell states, such as for AC-like marker gene *ITPKB* (Fig. 4i), which is linked to 11 CREs that harbor TF binding sites for SOX9, NFATC4 and RFX3, whose regulons are predicted to govern the expression of *ITPKB*. Together, our data further corroborate the closely interwoven and cell state-specific loops of chromatin regulation and gene expression identified at multiple levels.

In summary, we show that single-cell chromatin accessibility independently recapitulates the main cellular lineages identified in corresponding single-cell transcriptomes of H3-K27M DMG tumors. Our multimodal analysis reveals putative cell state-specific CREs as building blocks of larger GPC-associated regulatory complexes. These GPCs are enriched in OPC-like/OC-like cells, reinforcing the central role of the oligodendroglial lineage in H3-K27M DMGs. These results can be leveraged to more deeply investigate select key intrinsic regulators of H3-K27M DMG cell identities.

### The age-specific myeloid cell landscape in H3-K27M DMGs

Various cellular and structural components constitute the glioma microenvironment and extrinsically influence glioma cell identities^49,50^. It remains to be elucidated whether these components are characteristic of their respective location or age-related brain environments. Here our age- and location-stratified H3-K27M glioma cohort uniquely lends itself to dissecting such context-specific differences largely independent of tumor subtype and genetic drivers. Because glioma- or tumor-associated myeloid cells (GAMs/TAMs) presented the largest proportion of nonmalignant cells within our scRNA-seq dataset (Fig. 1c), we focused on characterizing and comparing this microenvironmental component across our clinico-anatomical patient groups.

**Fig. 5:**
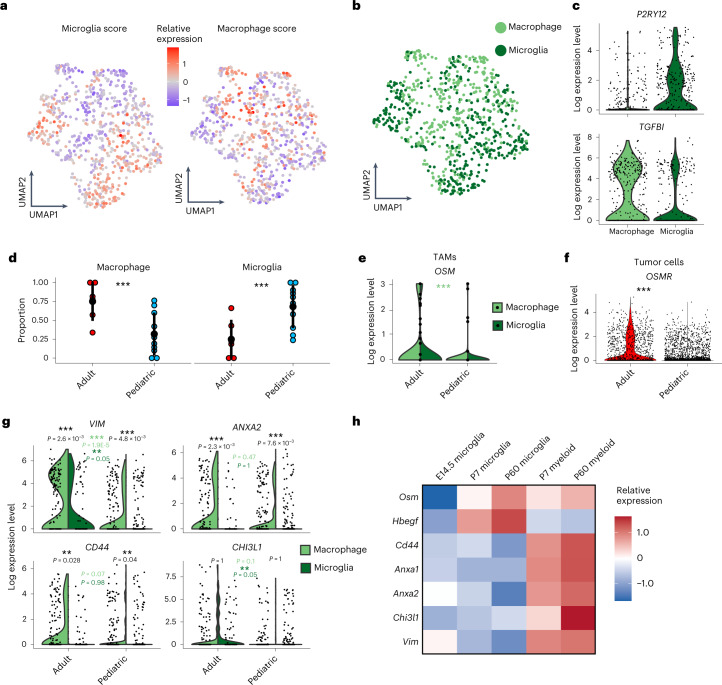
The myeloid cell landscape of H3-K27M DMGs. **a**, UMAP of TAMs analyzed by scRNA-seq, color scaled by expression scores for microglia and macrophage gene sets. **b**, UMAP of TAMs colored by classification as macrophage or microglia cell type. **c**, Violin plot depicting log normalized expression levels of representative microglia and macrophage marker genes across TAMs scored as either microglia or macrophage. **d**, Dotplots representing the distribution (mean ± 2 × s.e.m.) of assigned macrophage versus microglia proportions across adult and pediatric tumors (*N* = 16 biologically independent samples). Three asterisks denote credible statistical changes determined by a Bayesian scCODA model with FDR < 0.05 and without multiple test corrections. **e**, Violin plots of log normalized expression levels of *OSM* gene in adult and pediatric TAMs. Three asterisks denote *P* = 0.003 (two-sided Kolmogorov–Smirnov test). Three asterisks in light green represent comparisons between adult and pediatric tumors for macrophages. **f**, Violin plots of log normalized expression levels of *OSMR* gene in adult and pediatric tumor cells. Three asterisks denote *P* = 0 (two-sided Kolmogorov–Smirnov test). **g**, Violin plots of log normalized expression levels of MES-like marker genes in adult and pediatric TAMs. *P* values from different comparisons are shown (two-sided Kolmogorov–Smirnov tests; black: within age-group comparisons between macrophages and microglia; light green: adult versus pediatric macrophages; dark green: adult versus pediatric microglia). **h**, Heatmap representation of scaled relative expressions (color scale) of MES-like state-associated ligands and marker genes (rows) in a single-cell atlas of normal mice microglia and brain myeloid cells across different age groups (E14.5, P7, P60)^52^ (columns).

**Extended Data Fig. 5:**
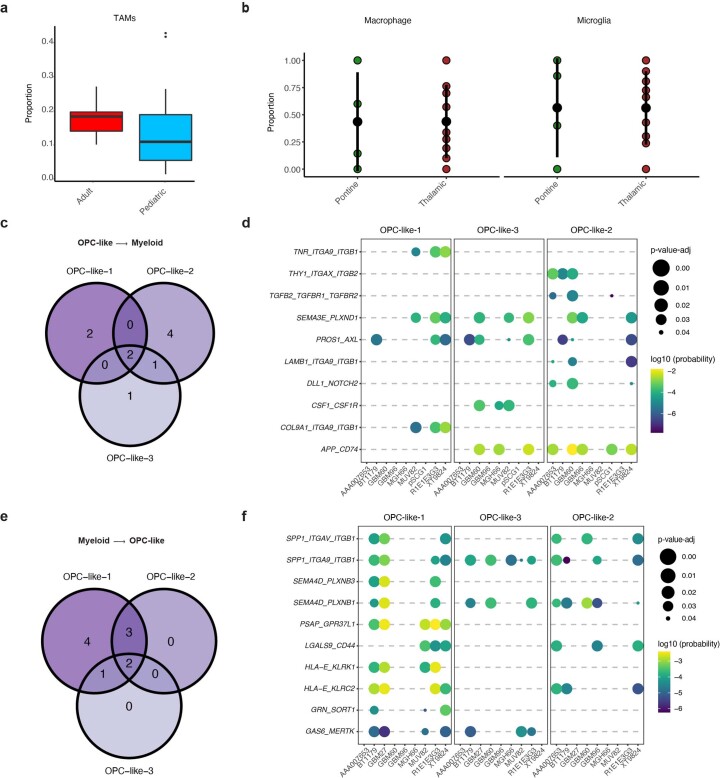
The myeloid cell landscape of H3-K27M DMGs. (a) Boxplot depicting TAM proportions in all tumor and normal cells profiled by scRNA-seq and grouped by adult and pediatric sample groups across N = 16 biologically independent samples. The median is marked by the thick line within the boxplot, the first and third quartiles by the upper and lower limits, and the 1.5x interquartile range by the whiskers. (b) Distributions (mean values + /− 2xSEM) of macrophage and microglia proportions within TAMs across N = 16 pontine and thalamic tumors. (c) & (e) Venn diagram depicting shared and specific OPC-like-to-myeloid (c) and myeloid-to-OPC-like (e) ligand-receptor interactions between different OPC-like subpopulations. (d) & (f) Ligand-receptor interactions assessed for each OPC-like subpopulation for OPC-like-to-myeloid (d) and myeloid-to-OPC-like (f) interactions. Color scale depicts probabilities of interaction, while dot size denotes Benjamini-Hochberg (BH)-corrected p-values from a two-sided permutation test.

We classified TAMs into brain-resident microglia or monocyte-derived macrophages using reported sets of canonical marker genes^35^ (Fig. 5a–c). Overall TAM proportions were not different between adult and pediatric samples (Extended Data Fig. 5a). However, comparison of microglia versus macrophage proportions across age groups revealed a higher rate of microglia in pediatric DMGs, while adult DMGs contained higher rates of macrophages (Fig. 5d). Tumor location did not seem to influence these proportions (Extended Data Fig. 5b).

Mounting evidence suggests a causal role of TAMs in establishing a mesenchymal cell state in GBM through TAM-secreted ligands binding to receptors on glioma cells, such as between ligand-receptor pair OSM-OSMR, or via chemokine signaling^27,28,40,51^. Given the significant enrichment of MES-like cells in adults compared to pediatric H3-K27M DMGs in our cohort, we hypothesized that this may be driven by differences in such tumor–immune interactions. We indeed detected higher expression of *OSM* in adult TAMs, and the corresponding receptor *OSMR* in adult tumor cells (Fig. 5e,f), indicating immune-mediated engagement of a previously validated pathway^27^ in inducing the MES-like phenotype in adult tumors. Moreover, we observed increased expression of MES-like marker genes in adults compared to pediatric TAMs, which were shown to be increased in mesenchymally enriched gliomas^27^ (Fig. 5g). To assess whether these transcriptional differences of MES-like state marker genes and inducing ligands may be inherent to normal brain myeloid cells during temporal development and aging, we analyzed gene expressions across age in a normal mouse brain myeloid cell atlas. Indeed, we observed an increase of ligands such as OSM and of mesenchymal marker genes with age (Fig. 5h)^52^, supporting that the increase of the H3-K27M DMG tumor MES-like state with age is linked to changes of the brain myeloid compartment that also occur during normal development and aging processes.

Last, we interrogated receptor–ligand interactions between TAMs and OPC-like subpopulations, revealing shared OPC-wide (for example, SEMA3E-PLXND1) and subpopulation-specific interactions (Extended Data Fig. 5c–f). This may point toward a harnessing of microenvironmental factors in reinforcing the OPC-like lineage and further determining their varying maturation, which provides the basis for follow-up investigations to better understand the contributions of cell-extrinsic regulators to the different OPC-like states.

In summary, we reveal that adult H3-K27M DMGs harbor higher proportions of monocyte-derived macrophages, while pediatric tumors are enriched for brain-resident microglia. We also show that H3-K27M DMG-associated TAMs upregulate ligands and marker genes that can induce tumor cell MES-like states with increasing age, thereby linking the age-specific tumor immune microenvironment to the observed increase of MES-like tumor cells in adult H3-K27M DMGs. This illustrates how age-related microenvironmental factors can differentially shape tumor cellular states.

### Charting the single-cell spatial architecture of H3-K27M DMG

**Fig. 6:**
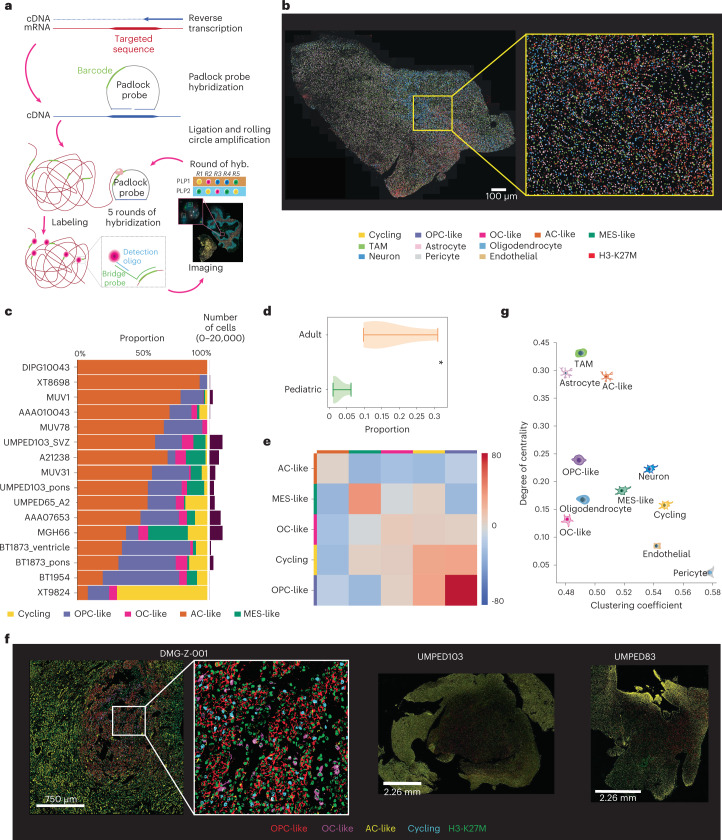
The single-cell spatial transcriptomic architecture of H3-K27M DMGs. **a**, Schematic of HybISS experimental approach. Briefly, mRNA is amplified in situ by RT, and the product cDNA is hybridized with a custom complementary padlock probe. Next, RCA reaction is run to generate a blob of DNA that can then be barcoded with individualized gene bridge probes and fluorescently barcoded. After imaging, the sample is stripped of bridge probes, and the cycle is repeated five times with different fluorophores for decoding and identification of gene signals based on their decoding sequence. **b**, Representative image of malignant and nonmalignant cell type/state assignments in one primary human H3-K27M DMG section (UMPED65_A2; 1 experiment over the entire tumor section with *N* = 22,813 cells assigned), outlining the distribution of malignant and nonmalignant cell populations within the sample. **c**, Proportions (*x* axis) of scRNA-seq derived tumor cell states (color legend) identified by pciSeq across 16 human H3-K27M DMG samples (*y* axis). **d**, Violin plot representing the distribution of MES-like cell proportions in adult compared to pediatric H3-K27M DMGs (*N* = 7,004 MES-like cells across 16 biologically independent samples) profiled by spatial transcriptomics. Whiskers show minimum/maximum proportions. An asterisk denotes *P* = 0.024 (two-sided *t*-test). **e**, Heatmap representations of neighborhood enrichment analysis between malignant cell populations, identified at 50 μm, across all samples. The color scale denotes the probability of finding a cell when a second cell type is presently divided by the probability of finding the second cell type. **f**, Representative multiplexed IF CODEX images from three of four primary human H3-K27M DMGs, showing spatially distinct subpopulations of malignant (marker: H3-K27M) OPC-like (marker: PDGFRA), OC-like (marker: BCAS1), AC-like (marker: GFAP) and proliferating cells (marker: Ki67). For each tumor, one experiment was performed with ~70,000 to 1.2 million individual cells profiled per sample over the entire tumor section. **g**, Sample-wide scatter plot representing each cell population’s tendency to cluster with other cell populations (degree of centrality, *y* axis) or to cluster with themselves (clustering coefficient, *x* axis).

**Extended Data Fig. 6:**
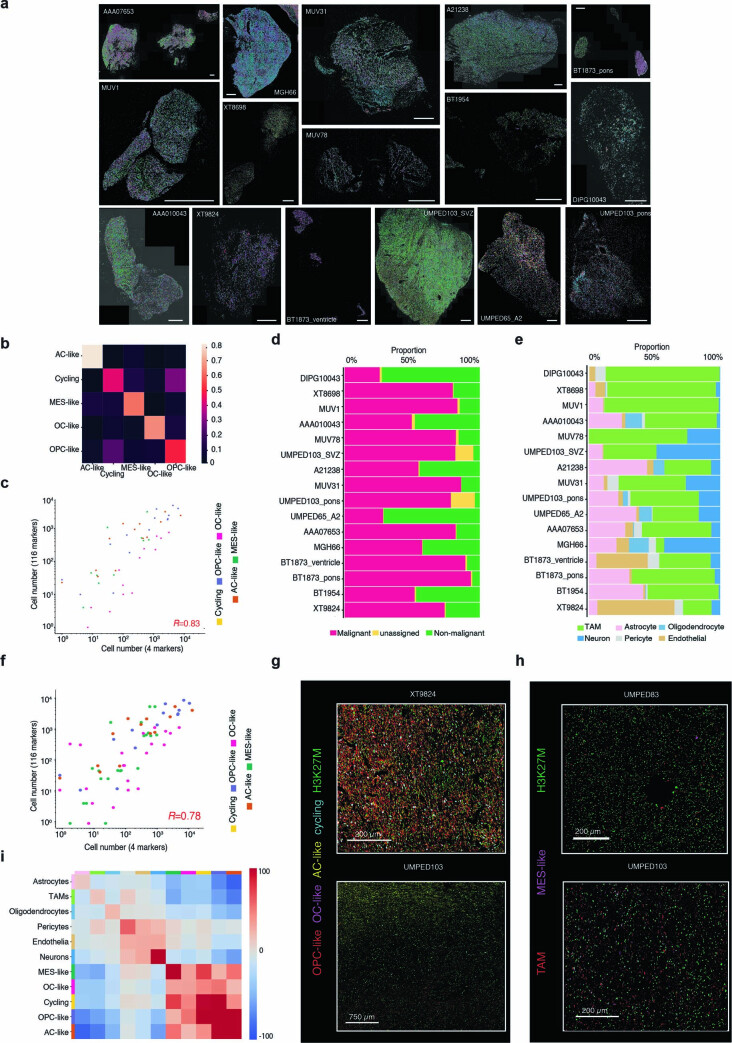
The single-cell spatial transcriptomic architecture of H3-K27M DMGs. (a) Representative HybISS gene maps for 16 H3-K27M tumors (1 experiment/tumor over the entire image section with 100-20,000 cells profiled/tumor). Scale bar corresponds to 100 µm in all panels. (b) Confusion matrix of pciSeq derived tumor cell state scores for all samples. The color scale represents the mean probability assigned to a cell when a specific cell state is predicted. Higher values indicate a more probable prediction. (c) Scatter plot representing numbers of malignant cells assigned to a cell state (color scale) for each sample (dot), as inferred from pciSeq based on 116 marker genes (y-axis) or on the 4 best markers (x-axis). The Pearson correlation coefficient between both marker sets is shown in red. (d) Sample-level proportions (x-axis) of malignant and non-malignant cells (color legend) across 16 tumors (y-axis) profiled by HybISS as assessed by anti-H3.3K27M IF. (e) Sample-level proportions (x-axis) of non-malignant cell types (color legend) assigned by HybISS for the 16 H3-K27M DMGs (y-axis). (f) Scatter plot representing numbers of malignant cells assigned to a specific cell state (color scale) for each sample profiled (dot), as inferred from pciSeq based on 116 marker genes (y-axis) or on selected IF markers (PDGFRA, BCAS1, GFAP, CD44/CD63) (x-axis). The Pearson correlation coefficient between both marker sets is shown in red. (g) & (h) Representative multiplexed IF (CODEX) images, showing spatially distinct subpopulations of malignant (marker: H3-K27M) OPC-like (marker: PDGFRA), OC-like (marker: BCAS1), AC-like (marker: GFAP), and proliferating cells (marker: Ki67) in (g), and of MES-like (marker: CD44/CD63) and myeloid cells (marker: IBA1) in (h). For each tumor, one experiment was performed with ~70,000-1.2 million individual cells profiled per sample over the entire tissue section. (i) Neighborhood enrichment analysis between all malignant and non-malignant cell populations, identified at 50 μm. The color scale denotes the probability of finding a cell when a second cell type is present divided by the probability of finding the second cell type.

To map our scRNA-seq/snATAC-seq derived cell populations to their spatial positions within intact H3-K27M DMG tissues, we performed hybridization-based in-situ sequencing (HybISS)^53^ in 16 patient H3-K27M DMG tissue sections (14 different tumors, 2 tumors with multi-region sampling), using a panel of 116 cell-type-specific combinatorial marker genes curated from our scRNA-seq dataset (Fig. 1b, 6a–c, Extended Data Fig. 6a–e and Supplementary Table 5).

We analyzed spatial cell state compositions by probabilistic cell typing by in situ sequencing (pciSeq). Here we interestingly observed AC-like cells to constitute the major malignant cell compartment (Fig. 6c), which is in contrast to the predominance of OPC-like cancer cells observed by scRNA-seq. This held true across tumor sections of different sizes, cell densities and qualities. Our spatial analysis also identified larger numbers and diversity of nonmalignant cell types, that were either not detected or showed only low representation in scRNA-seq (Extended Data Fig. 6e). Because larger numbers of cells are assessed on average, and processing-associated biases are reduced in intact tissues, spatial transcriptomics is likely more representative of true cell state compositions than conventional scRNA-seq. Stratification within our spatially profiled cohort again revealed that adult H3-K27M DMG sections harbor substantially higher proportions of MES-like tumor cells relative to pediatric tumors (Fig. 6d), orthogonally underscoring the association of age with the MES-like state.

We next performed neighborhood enrichment analyses to investigate spatial relationships between individual cell populations. Here we observed marked variability in neighborhood structures, highlighting overall intertumoral spatial heterogeneity (Supplementary Fig. 3). Global analysis of malignant cell neighborhoods indicated higher colocalization of OPC-like/cycling and OC-like cells (Fig. 6e). We validated these findings on the protein level by multiplexed immunofluorescence (IF) imaging (codetection by indexing (CODEX) system) in four H3-K27M gliomas (Fig. 6f and Extended Data Fig. 6f–h). Concordantly, this approach indicated a preferred mitotic niche of proliferating OPC-like and OC-like cells, encircled by more differentiated, nonproliferating AC-like cells (Fig. 6f).

Neighborhood analysis between cancer and noncancer cells revealed closer proximities between vascular cells and MES-like tumor cells (Extended Data Fig. 6i), pointing toward increased vascularization that has been associated with the mesenchymal state^54^. Within a subset of samples (7 of 12 with >1,000 cells profiled), we also observe increased colocalization of microglia/macrophages with MES-like, OC-like and AC-like cancer cells (Supplementary Fig. 3).

Further, we assessed the tendencies of each cell population to either form their own homogeneous cluster, by calculating their clustering coefficient (that is, degree to which members of a cell population favor clustering together), or to cluster heterogeneously with other populations, as represented by their degree centrality (that is, ratio of nonmembers connected to members of a cell population). Here we observed that AC-like cells, nonmalignant astrocytes and TAMs depicted the highest tendency to cluster with other cell types/states, hinting at their more diffuse distribution rather than localization within a restricted spatial compartment. In comparison, vascular cells, neurons, and cycling OPC-like cells exhibited a higher tendency to cluster with members of the same cell population, which is further indicative of a propensity to form specific structures/niches (Fig. 6g).

In summary, we resolved the spatial architecture of scRNA-seq–defined H3-K27M DMG cell populations directly within the native tumor tissue. Our results shed light on global and heterogeneous cellular relationships and neighborhoods, notably suggesting the presence of mitotic stem-like niches in which H3-K27M tumor cells of oligodendroglial lineage (OPC-like and OC-like cancer cells) colocalize. These findings lend themselves to further investigation of potential therapeutic avenues directed at regional and temporal perturbation of H3-K27M DMG tumor cell populations and their associated niches.

## Discussion

We previously demonstrated the preponderance of OPC-like tumor cells in seven pediatric H3-K27M DMGs through scRNA-seq. However, it remained unknown whether the same cellular composition—proposed to arise as a function of early pontine development—holds true across multiple spatiotemporal environments in which these tumors occur. To address these questions, we generated a multi-omic single-cell atlas of H3-K27M DMGs, comprising various midline locations and ages ranging from 2 to 68 years. Our data shed light on understudied thalamic locations and adolescent/adult age groups and provide a blueprint for the spatiotemporal context-specificity of tumor cell-intrinsic properties, spatial tissue architectures, and microenvironmental interactions that co-orchestrate cellular identity against the backdrop of the shared K27M driver mutation.

Our study reveals a ubiquitous presence of OPC-like and more differentiated glia-like cells across all clinico-anatomical groups. Concomitantly, neuronal-like tumor cells are absent, which is independent of age and location and stands in contrast to other glioma types. Thus, this likely presents direct consequences of the K27M mutation universally skewing tumor cells toward an OPC-like and away from a neuronal-like state, decoupled from spatiotemporal influences.

**Fig. 7:**
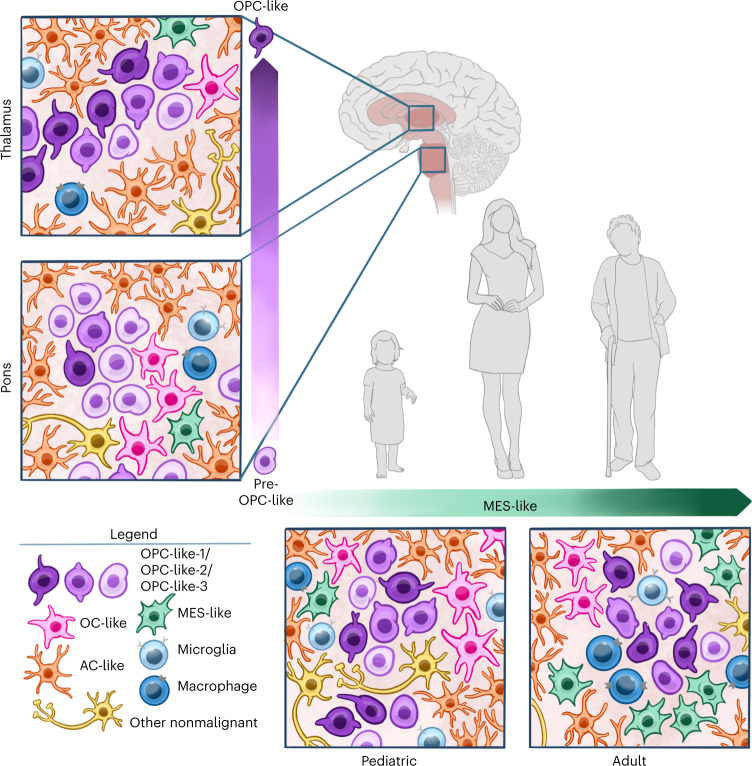
Schematic summary of the spatiotemporal context-specific composition of H3-K27M DMGs. Comparisons are between pediatric versus adult patient groups (*x* axis) and pontine versus thalamic midline locations (*y* axis) and a representative model image of tumor cell composition is depicted, respectively. All tumor groups are abundant in OPC-like cells and also harbor more differentiated AC-like, OC-like, MES-like and nonmalignant microenvironmental cells, but lack tumor cells of the NPC/neuronal lineage, as delineated by single-cell multi-omics (color legend). MES-like cells increase with age, as indicated by the green arrow, which is associated with age-related changes in the tumor immune microenvironment; in particular, higher proportions of microglia in pediatric tumors as opposed to increased proportions of macrophages in adult tumors. Location specificity exists for varying maturation stages of OPC-like cells—pontine tumors harbor less mature pre-OPC-like cells, while thalamic tumors are enriched for more mature lineage-committed OPC-like cells, either as a result of region-specific cell-intrinsic features or due to location-related diversification driven through interactions within the local environmental niche.

We identify two major variable features as a function of regional or temporal context, respectively (Fig. 7):

First, we resolve pontine H3-K27M DMGs to harbor more immature pre-OPC-like tumor cells than their thalamic counterparts. This raises the question of whether this diversity reflects region-specific cell-intrinsic features or it is driven by local environmental interactions. While normal murine OPCs have been shown to lack heterogeneity across different brain regions^44,55^, it is possible that region-specific microenvironments provide distinct cues to differentially foster OPC differentiation. This has been observed in the gray matter where OPC differentiation takes place more slowly compared to white matter^56,57^. In glioblastoma, the white matter has likewise been suggested as a prodifferentiative niche for oligodendroglial lineage stem-like cells^58^. It will be of interest to explore in future studies what extrinsic factors in the pons relative to the thalamus may contribute to preserving healthy and aberrant OPC(-like cell)s in a less committed pre-OPC(-like) state and how these specific microenvironmental contexts could be perturbed by targeting such factors.

The finding of a more immature precursor-like cell is accordant with previous modeling studies postulating embryonic neural stem/progenitor cells instead of OPCs as the H3-K27M DMG cell of origin^11,59–62^. While the K27M mutation could occur in such an earlier state, it subsequently induces a cellular arrest in a self-renewing OPC-like state^59^, and the hypothesized original cell of mutation may become diluted and eliminated from fully transformed tumors^9^. Taken together, the literature supports the idea that the cell state of transformation is an oligodendroglial lineage precursor, whose precise state may vary from pre-OPC to more mature OPC with different histone variants^19^, anatomical locations and ages.

Second, we observe the mesenchymal signature to increase with higher age, which we link to age-related differences in TAMs that have been illustrated to induce this myeloid-affiliated tumor signature^27,28,40^. As the mesenchymal state has been associated with a more aggressive phenotype in a broad range of solid tumors^63,64^, and mesenchymal- and myeloid-directed therapies are under active investigation, it will be of interest to investigate such an age and outcome association in H3-K27M gliomas and other tumors.

Lastly, we reconstructed the single-cell spatial architecture of patient H3-K27M tumors, identifying a niche of proliferating OPC-like/OC-like tumor cells, surrounded by AC-like cells, which constitute the major tumor cell population in situ. This finding contrasts the predominance of OPC-like cells observed by conventional and especially fresh scRNA-seq and may arise due to technical and biological reasons. As AC-like glioma cells have been shown to be interconnected through tumor microtubes^19,25,65^, we speculate that they may be less viable and more sensitive to tumor dissociation, thereby biasing toward capturing more aggressive OPC-like cells in scRNA-seq. By contrast, AC-like cells may be better preserved in frozen snRNA-seq and spatial approaches. Such a potential predominance of AC-like cells instead of OPC-like cells does not stand in contrast to the proposed role of OPC-like cells as the stem-like drivers of H3-K27M DMGs and would align with a more traditional model in which cancer stem cells present the minority of tumor cells^66^. With the emergence of spatial technologies, it will be relevant to assess whether similar differences are observed throughout other tumor types and biological systems, pinpointing the importance of multimodal profiling to further refine models derived primarily through the lens of a single modality.

Altogether, we provide an extensive resource of H3-K27M DMG cellular heterogeneity across space and time that lends itself to delineating the multi-faceted interplay between spatiotemporal context-specific cellular properties and microenvironmental niches for the design of rational modeling studies and therapeutic frameworks tailored to the different clinico-anatomical groups of this lethal glioma.

## Methods

### Human subjects and ethical considerations

All samples used in this study were deidentified and obtained with properly informed consent of patients and/or their legal representatives, who did not receive compensation. The study was approved by the Institutional Review Board at Boston Children’s Hospital/Dana-Farber Cancer Institute (DFCI 10-417) and at affiliated research hospitals or via waiver of consent as appropriate. Clinical information (age, sex and location) and mutation status are presented in Fig. 1b and Supplementary Table 1.

### Tumor tissue collection and dissociation

Fresh tumor tissue acquired at the time of surgery was immediately mechanically and enzymatically dissociated for 30 min at 37 °C using the Brain Tumor Dissociation Kit (Miltenyi Biotec). Single-cell suspensions were filtered through a 70 µm strainer, centrifuged at 500*g* for 5 min, and resuspended in PBS/1% BSA for fluorescence-activated cell sorting (FACS).

To extract single nuclei from frozen tissues for snRNA-seq, snap-frozen or OCT-embedded tumor tissue was disaggregated on ice in 1 ml 0.49% CHAPS detergent-based nuclear extraction buffer^67^, aided by mild chopping. Single-nuclei suspensions were filtered using a 40 µm strainer and centrifuged at 500*g* for 5 min. All steps were performed at 4 °C.

To prepare single-nuclei suspensions for snATAC-seq, snap-frozen DMG tissue was lysed on ice in lysis buffer (10 mM Tris–HCl, 10 mM NaCl, 3 mM MgCl_2_, 1% BSA, 0.01% Tween-20, 0.01% NP-40, 0.001% digitonin) under mild chopping for 5 min, followed by ten times mixing using a wide-bore pipette tip and 10 min incubation on ice. Wash buffer (10 mM Tris–HCl, 10 mM NaCl, 3 mM MgCl_2_, 1% BSA, 0.1% Tween-20) was added and mixed five times before filtering through 70 and 40 µm Flowmi cell strainers. Single-nuclei suspensions were then centrifuged at 500*g* for 5 min at 4 °C, resuspended in 1× diluted nuclei buffer and counted.

### scRNA-seq/snRNA-seq data generation

Whole transcriptome amplification, library preparation and sequencing of single cells/nuclei were performed using the Smart-seq2 modified protocol^21,33,35,68,69^. RNA was purified with Agencourt RNAClean XP beads (Beckman Coulter). Oligo-dT primed reverse transcription (RT) was performed using Maxima H Minus reverse transcriptase (Life Technologies) and a template-switching oligonucleotide (TSO; Qiagen). PCR amplification (20 cycles for scRNA-seq and 22 cycles for snRNA-seq) was performed using KAPA HiFi HotStart ReadyMix (KAPA Biosystems), followed by Agencourt AMPure XP bead (Beckman Coulter) purification. Libraries were generated using the Nextera XT Library Prep kit (Illumina). Libraries from 768 cells with unique barcodes were combined and sequenced using a NextSeq 500/550 High Output Kit v2.5 (Illumina).

### scATAC-seq data generation

scATAC-seq libraries were generated using the 10X Chromium Controller and Chromium Next GEM Single Cell ATAC & Library Gel Bead Kit v1.1 kit according to the manufacturer’s instructions (Document CG000209). Briefly, 7,000–10,000 nuclei were tagmented at 37 °C for 60 min and loaded on a Chromium Next GEM Chip H and Chromium Controller for generation of single-cell Gel Bead-In-Emulsions, followed by linear amplification of barcoded tagmented DNA. GEMs were then broken up, DNA fragments were purified using Dynabeads MyOne SILANE (10X 2000048) and SPRIselect Reagent (Beckman Coulter, B23318), and further PCR-amplified for 10–11 cycles undergoing sample indexing. Libraries were sequenced using a NextSeq 500/550 High Output Kit v2.5 (Illumina) at targeted 25,000 reads per cell.

### Gene selection for targeted HybISS

Gene panel selection was based on the scRNA-seq data from ten H3-K27M DMG patient tumors spanning multiple clinico-anatomical groups and on published datasets of normal brain-resident cell types^70^ (Supplementary Table 5). Genes were prioritized based on differential expression between cell types, followed by manual filtering of genes with likely high background expression levels being strongly expressed in all cell types. A total of 618 probes were designed for 116 genes encompassing malignant (OPC-like, AC-like, OC-like and MES-like) and nonmalignant cells (oligodendrocytes, astrocytes, neurons, macrophages, microglia, T cells, endothelia, pericytes and ependymal cells) (Supplementary Note 1).

### HybISS

After fixation with 3% PFA for 30 min, sections were permeabilized with 0.1 M HCl and washed with PBS. After rehydration for 1 min in 100% ethanol, 1 min in 75% ethanol and 1 min in PBS, cDNA was synthesized overnight with reverse transcriptase (BLIRT), RNase inhibitor, and primed with random decamers. Sections were postfixed before padlock probe (PLP) hybridization and ligation at a final concentration of 10 nM/PLP, with Tth Ligase and RNaseH (BLIRT). This was performed at 37 °C for 30 min and then 45 °C for 1 h. Sections were washed with PBS, followed by rolling circle amplification (RCA) with phi29 polymerase (Monserate) and Exonuclease I (Thermo Fisher Scientific) overnight at 30 °C. Bridge probes (10 nM) (Supplementary Table 6) were hybridized at RT for 1 h in hybridization buffer (2× saline- sodiumcitrate buffer (SSC), 20% formamide), followed by hybridization of readout detection probes (100 nM) and DAPI (Biotium) in hybridization buffer for 1 h at RT. The sections were washed with PBS and mounted with SlowFade Gold Antifade Mountant (Thermo Fisher Scientific). After each imaging round, coverslips were removed and sections were washed five times with 2× SSC. Bridge probe/detection oligonucleotides were then stripped with 65% formamide and 2× SSC for 30 min at 30 °C, followed by five washes with 2× SSC. The above procedure was repeated for cycles 1 through 5, leading to hybridization of cycle-specific individual bridge probes (for imaging, see Supplementary Note 1).

### CODEX

FFPE tissue sections were collected onto poly(l-lysine)-coated coverslips and prepared according to the Akoya Biosciences CODEX protocol^71^. Sections were then deparaffinized and rehydrated. Antigen retrieval was performed using a pressure cooker and 1× citrate buffer, pH 6.0. Sections were then quenched for autofluorescence^72^, and subsequently stained and imaged using the Akoya Biosciences CODEX staining kit (7000008). Tissue was stained using the following preconjugated antibodies purchased from Akoya: DAPI (7000003), Ki67-BX047 (B56)—Atto 550-RX047 (4250019) 1:200, CD44-BX005 (IM7)—Atto 550-RX005 (4250002) 1:50. The following antibodies were custom conjugated using the Akoya Biosciences conjugation kit (7000009) and indicated barcodes: anti-PDGFRα antibody (Abcam, ab234965) Barcode BX002—Atto 550-RX002 (5450023) 1:50, anti-BCAS1 antibody (Santa Cruz Biotechnology, sc-136342) Barcode BX027—Cy5-RX027 (5350004) 1:50, anti-GFAP antibody (Invitrogen, 13-0300) Barcode BX030—Cy5-RX030 (5350005) 1:50, antihistone H3 (mutated K27M) antibody (Abcam, ab240310) Barcode BX004—Alexa FluorTM 488-RX004 (5450014) 1:100, anti-IBA1 antibody (Thermo Fisher Scientific, GT10312) Barcode BX020—Atto 550-RX020 (5250002) 1:50, anti-CD63 antibody (353039, Biolegend) Barcode BX029—Atto 550-RX029 (5250005) 1:50. Imaging was performed using a Keyence BZ-X800E fluorescent microscope equipped with a BZ Nikon Objective Lens (×20). Images were processed using the CODEX processor software (Akoya) and visualized using the ImageJ plugin CODEX Multiplex Analysis Viewer.

### Statistics and reproducibility

No statistical method was used to predetermine the sample size. No data were excluded from the analyses. The experiments were not randomized. Data collection and analysis were not performed blind to the conditions of the experiments.

Statistical analysis was performed in R v.4.0.3. A Bayesian statistical framework scCODA (v0.1.4) was used to identify changes in the proportion of different cell populations between age groups and anatomical departments. Comparisons of numerical variables between different conditions were carried out using Wilcoxon rank-sum test and Kolmogorov–Smirnov test, as appropriate. Overlap between groups of genes was assessed using a Hypergeometric test.

Single-cell sequencing for each tumor was performed in one experimental replicate. This is typical for human studies because tissues are usually limited and cannot be analyzed more than once. At least three samples per age and anatomical group were collected to verify reproducibility. The ISS and IF experiments for each tumor sample were performed in one experimental replicate, where the entire section was imaged. For RNAish experiments, two to three slides were stained per sample and approximately 10–15 fields of view were captured per slide. Further information on research design is available in the Nature Research Reporting Summary.

### scRNA-seq data processing

We aligned raw sequencing reads to hg19 genome by hisat2 (v2.1.0) and quantified and normalized gene counts using RSEM (v1.3.0) as transcript-per-million/TPM^73^. For snRNA-seq data, we modified the gene annotation files to count introns^74^. We calculated expression levels as *Ei*,*j* = log_2_(TPM*i*,*j*/10 + 1) for gene *i* in sample *j*. To filter out low-quality cells in fresh samples, we removed cells with <2,000 genes or an average housekeeping gene expression of <2.5. For frozen tumors, a filtering threshold of <1,000 genes and an alignment rate of <0.4 were employed. In sum, 9,911 high-quality cells were retained. We also removed genes with TPM > 16 in <10 cells. For the remaining cells and genes, we computed the aggregate expression of each gene as Ea(*i*) = log_2_(average(TPM*i*,1…*n*) + 1) and defined relative expression as centered expression levels, Er*i*,*j* = E*i*,*j* − average(E*i*,1…*n*). On average, we detected 6,866 uniquely expressed genes per cell in fresh, and 4,432 uniquely expressed genes in frozen tumors.

### Data harmonization, Louvain clustering and identification of differentially expressed genes

Graph-based clustering with data integration was adapted for independent identification of cellular clusters and gene signatures. We selected highly variable genes (HVGs) using Seurat (v3.2.2)^75^ and used the relative expression values of these HVGs for PCA. To disentangle sample-specific biological variations (that is, tumor-specific genetic and epigenetic alterations) from cell subpopulation-specific variations and to integrate multiple samples, we applied a linear adjustment method (Harmony v1.0) to the first 100 PCs with default parameters to generate a corrected embedding^76^. We chose the first 20 Harmony-corrected dimensions for uniform manifold approximation and projection embedding (UMAP) embeddings, and clustered cells by Seurat’s Louvain algorithm-based FindClusters function. Cells from different samples expressing similar gene programs were well mixed (Extended Data Fig. 2a). We next identified differentially expressed genes by Seurat’s FindAllMarkers function. We tested genes that were detected in a minimum of 30% of the cells within each cluster and that showed at least a 0.5-fold mean log difference. We utilized Wilcoxon rank-sum test with Bonferroni correction for multiple testing and only kept genes with adjusted *P* value < 0.05.

### Nonnegative matrix factorization (NMF) metaprogram analysis

NMF was used to assemble transcriptional programs from relative expressions (with negative values converted to zero)^21,68,69^. We derived NMF programs for malignant cells from each sample using the top 10,000 over-dispersed genes, as determined by PAGODA2 (v0.1.4)^77^. The number of factors was set to six for each sample. Because redundant NMF programs were merged into a single metaprogram, the final metaprogram was not sensitive to the initially chosen number of factors. We selected the top 30 genes with the highest NMF weights from each NMF factor and scored all malignant cells with these NMF programs. We then clustered NMF programs by hierarchical clustering (distance metric: 1 − Pearson correlation; linkage: Wardʼs linkage) on the scores for each NMF program (Extended Data Fig. 2b). This revealed eight highly correlated sets of programs in fresh tumors and nine in frozen tumors. We merged these correlated programs into metaprograms by selecting the top 30 genes with the highest average NMF weight within each correlated program set (Supplementary Table 2 and Supplementary Note).

### Comparison and integration of fresh and frozen tumor metaprograms

We compared transcriptional metaprograms independently derived from fresh and frozen tumors by pairwise correlation analysis, showing high correlations between the cycling, fresh OPC-like-1/frozen OPC-like-a, OC-like, AC-like and MES-like signatures (Extended Data Fig. 2d). Even though ribosomal protein-encoding genes marking the fresh OPC-like-2 metaprogram were filtered out in the frozen dataset to exclude potential technical artifacts from random capture of nuclei-associated ribosomes^67^, the frozen OPC-like-b program showed high correlation with the fresh OPC-like-2 signature (Extended Data Fig. 2d) and showed higher expression of pre-OPC markers, such as *DLL1, HES6* and *EGFR* (Supplementary Fig. 2). Therefore, we independently identified pre-OPC-like cells in our fresh and frozen scRNA-seq/snRNA-seq data. We consequently scored frozen nuclei for all fresh metaprograms, only exchanging fresh OPC-like-2 with frozen OPC-like-b to avoid artifacts due to the filtering of ribosomal protein genes. If the resulting maximum expression score was <0.2, single nuclei were classified as ‘score_too_lowʼ; if ≥0.2, nuclei were assigned according to the highest-scored metaprogram (Extended Data Fig. 2e,f).

### Analysis of cell type compositions

We applied the Bayesian model-based single-cell compositional data analysis (scCODA v0.1.4) framework to identify associations of cell compositions with different clinical covariates^78^. scCODA employs hierarchical Dirichlet-multinomial distribution that accounts for the uncertainty and negative correlative bias in compositional analysis of cell type proportions. The model uses a logit-normal spike-and-slab prior with a log-link function and Hamiltonian Monte Carlo sampling to estimate the effects of covariates on cell type proportions. The sample level counts of cell annotations and clinical covariates were used as inputs for scCODA. The default parameter was used with AC-like cells selected as the reference cell type. Locations and ages were included as covariates in the model. The statistical significance of changes in cell compositions was assessed using credible effects with a 5% false discovery rate.

### snATAC-seq data processing

Cell Ranger ATAC (v1.0.1) was used to process 10X Chromium snATAC-seq data. We used cellranger-atac counts to generate single-cell accessibility counts and cellranger-aggr to aggregate multiple samples without setting any normalization. The resulting peak-cell matrix and metadata were then analyzed in Signac (v1.1.0)^79^.

We removed nuclei with <200 detected peaks and peaks detected in <10 nuclei. We further kept nuclei with the following: (1) total number of fragments in peaks (peak_region_fragments) between 1,500 and 15,000, (2) percent of reads in peaks (pct_reads_in_peaks) >15, (3) ratio of reads in genomic blacklist regions (blacklist_ratio) <0.02, (4) approximate ratio of mononucleosomal to nucleosome-free fragments (nucleosome_signal) <2 and (5) ratio of fragments centered at the transcription start site (TSS) to fragments in TSS-flanking regions (TSS_enrichment) >4. After quality control and filtering, a dataset comprising 211,096 peaks and 9,797 nuclei was used for downstream analysis.

We normalized data using term frequency-inverse document frequency (RunTFIDF) and conducted dimensionality reduction using singular value decomposition and top 25% of features. We calculated k-nearest neighbors using FindNeighbours (reduction = ‘lsiʼ, dims = 2:30) and omitted the first latent semantic indexing (LSI) component as it exhibited a strong correlation with sequencing depth. We then identified cell clusters by shared nearest neighbor modularity optimization-based clustering algorithm and ran the FindClusters function (algorithm = 3/SLM and resolution = 0.8), and generated a UMAP embedding using the RunUMAP function with 2–30 LSI components.

We calculated gene activities for each gene in each nucleus by summing the peak counts in the gene body + promoter region (2 kb upstream of TSS). We then normalized gene activities to the median of total gene activities and performed log transformation. Genes with differential activities (DAGs) were identified by running FindAllMarkers on normalized gene activities. We tested genes that were detected in a minimum of 20% of the cells within each cluster by Wilcoxon rank-sum test with Bonferroni multiple test correction and only kept genes with log fold change >0.1 and adjusted *P* < 0.05. Top DAGs were used for initial annotation of each cell cluster. Putative nonmalignant clusters with highly accessible canonical marker genes were identified, including microglia (for example, *CD14*, *CSF1R* and *SPP1*), T cells (for example, *CD2*, *CD3D* and *RHOH*) and tumor-associated oligodendrocytes (for example, *BCAS1*, *SOX10* and *SIRT2*).

### scRNA-seq/snATAC-seq data integration

We applied canonical correlation analysis as implemented in Seurat to integrate log normalized gene activity scores of ATAC-seq data and gene expression scores of RNA-seq data. We used Seurat’s ‘FindTransferAnchorsʼ function for integration, specified the union of the 2,764 and 2,000 most variable genes in scRNA-seq and snATAC-seq respectively as input features, ‘ccaʼ as the reduction method, and default values for the rest of the parameters. For each cell profiled by snATAC-seq, we identified the nearest neighbor cell in those profiled by scRNA-seq with a nearest-neighbor search in the joint canonical correlation (CCA) L2 space. Nearest neighbors were determined by the ‘FNNʼ R package with the ‘kd_treeʼ algorithm.

### Linking gene regulatory elements and gene expression across all cell types

Because RNA expressions and chromatin accessibilities were measured in different cells, we applied a correlation-based approach to pseudobulk samples aggregating snATAC-seq and scRNA-seq counts from computationally matched cells to identify peak-to-gene links as putative CREs. We defined pseudobulk samples by randomly sampling 200 cells from the snATAC-seq dataset and combined each of these 200 seed cells with their respective 99 nearest neighbor cells in the Harmony-corrected ATAC-LSI space. Hence, each of the resulting pseudobulk sample comprised 100 cells. We computed pseudobulk peak counts by summing peak counts across respective counts of all 100 cells within each pseudobulk sample. Within each pseudobulk, we matched 100 ATAC cells with 100 RNA cells as their nearest neighbors in CCA L2 space and obtained pseudobulk RNA gene counts by summing gene counts across the respective counts of all 100 cells within each pseudobulk sample. Pseudobulk gene counts were normalized as TPM.

We then defined putative peak-gene pairs by associating peaks with a genomic distance within 250 kb of the TSS of genes profiled by scRNA-seq. Each peak is only linked to its nearest gene. For each candidate peak-gene pair, we determined the Pearson correlation coefficient of peak counts (normalized as CPM) and gene expression (TPM), and adjusted *P* values for these coefficients from a *t*-statistic using Benjamini-Hochberg (BH) procedure. We identified a set of 13,632 high-confidence peak-to-gene links by only retaining pairs with |PCC|>0.2 and BH-adjusted *P* < 0.05.

### Integrative TF analysis

We integrated scRNA-seq and scATAC-seq data to identify putative regulatory networks of TF-target pairs. For each TF documented in the JASPAR (2020) TF motif database, we computed its mean expression (TPM) and examined the frequency of its motif(s) within the CREs located in the TSS ± 10 kb region of its predicted target genes by SCENIC^80^. We then kept TFs with mean TPM > 4 and over-represented binding motifs in CREs. Next, we kept TFs that were among the top 30 TF regulons with the highest specificity score of any cell type. This resulted in a total of 65 TFs (Supplementary Table 4). Of these TFs, 19 were specific to OPC-like cells (for example, EGR1, JUN, HES6), 10 were specific to OC-like cells (for example, SOX4, SOX10), 21 were specific to AC-like cells (for example, GLI2, STAT3 and SOX9) and 15 were specific to MES-like cells (for example, FOSL2, CEBPD and ELK3).

For each GPC, we leveraged two complementary approaches to identify core TFs that may regulate expressions of this gene. First, we selected TFs that were predicted to regulate expressions of the target GPC by SCENIC analysis. Second, we examined if TFs identified above possess binding motifs that are over-represented in the CREs linked to the target GPC using a hypergeometric test. We kept TFs that are predicted to govern the expression of a target GPC and harbor binding motifs substantially enriched in CREs linked to the target GPC (Supplementary Table 4).

### Analysis of HybISS data

Image processing and decoding. Each field of view (FOV) image was maximum intensity projected to obtain a flattened two-dimensional image. These images were then analyzed using in-house custom software that handles image processing and gene calling based on the python package Starfish v0.2.1 (ref. ^81^). Each two-dimensional FOV was exported, and preprocessed including alignment between cycles, and stitched together using the MIST algorithm. Stitching was followed by retiling to create smaller nonoverlapping 6,000 × 6,000 pixel images that were then used for decoding. The decoding pipeline can be found at https://github.com/Moldia/iss_starfish/. Using Starfish, images were initially filtered by applying a white top hat filter. The filtered images were subsequently normalized, and spots were then detected using the FindSpots module from Starfish and decoded using MetricDistance decoding.

### Malignant versus nonmalignant cell typing

To distinguish between malignant (H3-K27M positive) and nonmalignant (H3-K27M negative) cells, ISS expression maps were aligned to IF images, both taken from the same tissue section, and the mean IF intensity of each cell was calculated. All IF H3-K27M positive cells were categorized as malignant based on a minimum IF threshold in each sample, while DAPI positive and H3-K27M negative cells were categorized as nonmalignant based on a maximum IF threshold. Cells with IF intensities between the two thresholds were considered ambiguous and excluded from the analysis. We obtained spatial transcriptomic profiles of a total of 125,801 high-quality cells (56,664 malignant cells, 69,137 nonmalignant cells).

### pciSeq

To identify the cellular identity of nonmalignant and cancer cells, two different methods were applied. Probabilistic cell maps of malignant cells were created using pciSeq v0.0.45. The pciSeq pipeline assigns the spatial coordinates of genes from the ISS maps to DAPI-stained nuclei based on the proximity and assigns individual cells to cell type definitions defined by our H3-K27M DMG scRNA-seq dataset. The pciSeq pipeline is publicly available (https://github.com/acycliq/pciSeq)^82^. In contrast, due to the presence of uniquely expressed markers in the panel, nonmalignant cell types were identified by the expression of key marker genes in each sample. Here we assigned nonmalignant cell types by lack of H3-K27M signal in IF staining and concomitant expression of key markers, such as MBP for oligodendrocytes, ESAM for endothelial, MYL9 for pericytes, GFAP for astrocytes, CD74 for TAMs, DLG4 for neurons. T cells were excluded from downstream analyses due to very low numbers identified.

### Spatial enrichment and neighbors analysis

To explore proximities between the different cell types, neighborhood enrichment analysis was performed using Squidpy v1.1.2 (ref. ^83^). Briefly, the spatial coordinates of the mapped cells were used to identify spatial enrichment of cell types at a specific radius, and an enrichment score for each defined cell type was calculated based on the number of connections for each cell cluster. The number of observed connection events was compared against 100 permutations, and a *Z* score was computed for each cell type that can be positive (indicating positive colocalization) or negative (indicating negative colocalization). Centrality scores and clustering coefficients were calculated for all samples and each individual sample as previously indicated^83^. Degree centrality represents the fraction of nongroup members, establishing each cell type as a group, connected to the cells assigned to the cell type analyzed. The clustering coefficient represents the degree to which nodes in the graph tend to cluster together. It is formulated as the number of closed triplets, defining a triplet as three connected nodes, over the total number of triplets. Calculation of scores was implemented in SquidPy v1.1.2 (ref. ^83^).

### Reporting summary

Further information on research design is available in the Nature Portfolio Reporting Summary linked to this article.

## Online content

Any methods, additional references, Nature Portfolio reporting summaries, source data, extended data, supplementary information, acknowledgements, peer review information, details of author contributions and competing interests, and statements of data and code availability are available at 10.1038/s41588-022-01236-3.

## Supplementary information

Supplementary Information Supplementary Figs. 1–3 and Supplementary Note.

Reporting Summary

Supplementary Table Supplementary Tables 1–7.

## Data Availability

ScRNA-seq and scATAC-seq data of primary patient DMGs have been submitted to GEO (GSE184357). ISS data are available at Zenodo under ID 6805729. Previously published scRNA-seq data reanalyzed in this study are available under accession codes GSE102130 (ref. ^21^), GSE122871 (ref. ^43^), GSE144462 (ref. ^41^), GSE131258 (ref. ^46^) and GSE123030 (ref. ^52^). WES data generated in this study are deposited in EGA (EGAS00001006431). For targeted exome-sequencing data, the majority of which was generated as part of routine clinical care, variant data have been included as Supplementary Table 7 for all samples except for A21–238 and AAA010043 as these were generated by external care providers with restricted data access. Previously published WGS data of tumors ICGC-GBM27, ICGC-GBM96 and ICGC-GBM60 are deposited at EGA00001001139, and WGS data for BT836 and BT869 have been published under dbGaP accession number phs002380.v1.p1 (ref. ^84^). H3-K27M DMG ChIP-seq data were utilized from GSE126319 (ref. ^19^).

## Code availability

Custom scripts and code used in data processing and figure creation are available at 10.5281/zenodo.7073167 (ref. ^85^).
