# Supplementary material for: The landscape of tumor cell states and spatial organization in H3-K27M mutant diffuse midline glioma across age and location
Source: Nat Genet. 2022 Dec 5;54(12):1881–94. doi: 10.1038/s41588-022-01236-3 (PMC9729116; doi:10.1038/s41588-022-01236-3)
Supplement: Supplementary file 1 — Supplementary Figs. 1–3 and Supplementary Note. [file 41588_2022_1236_MOESM1_ESM.pdf]

nature genetics

Article

https://doi.org/10.1038/s41588-022-01236-3

The landscape of tumor cell states and spatial organization in H3-K27M mutant diffuse midline glioma across age and location

In the format provided by the authors and unedited

Supplementary Information

Supplementary Note

Fluorescence-activated cell sorting (FACS). Single-cell suspensions obtained from fresh tumors in PBS+1% BSA were stained with 0.5-1 µM calcein AM (Life Technologies, C3100MP) and 0.33 µM TO-PRO3 iodide (Life Technologies, T3605) for 15 min at RT and then kept on ice. Single-cell sorting was performed on a SH800 (Sony) sorter using 488 nm (calcein AM, 530/30 emission filter) and 633 nm (TO-PRO-3, 665/30 emission filter) lasers. Viable cells were identified by positive staining for calcein AM and negative staining for TO-PRO-3. Doublets were discerned based on back scatter area (BSC-A) versus back scatter width (BSC-W) (Supplementary Figure 1). Singlet viable cells were sorted into 96-well plates containing cold TCL buffer (Qiagen, 1031576), briefly spun down, snap frozen on dry ice, and stored at -80°C.

Single-nuclei suspensions extracted from frozen tumors were stained with 0.5 µM Vybrant DyeCycle™ Ruby Stain (Invitrogen, V10309) immediately before FACS. Intact nuclei were selected by positive staining for Ruby Stain on the SH800 sorter (633 nm laser, 665/30 nm emission filter). Doublets were excluded in the Ruby Stain area versus Ruby Stain height setting (Supplementary Figure 1). Singlet nuclei were sorted into 96-well plates containing TCL buffer and 1% beta-mercaptoethanol, briefly spun down, snap frozen on dry ice and stored at -80 °C.

WES sample and library preparation. Genomic DNA was extracted using the QIAamp DNA Micro Kit (Qiagen, 56304) following manufacturer's instructions. gDNA was fragmented to 200 bp on a Covaris M220 instrument. Libraries were prepared using Swift S2 Acel reagents on a Beckman Coulter Biomek i7 liquid handling platform from approximately 200 ng of DNA with

14 cycles of PCR amplification. Finished libraries were quantified by Qubit fluorometer and fragment size distribution was evaluated by Agilent TapeStation 2200. Five libraries were combined to a total amount of 1,500 ng (300 ng/library) and dried down in a vacuum concentrator with no heat. Dried indexed library pools were resuspended using Twist Biosciences reagents and hybrid capture was performed with a 16 hour hybridization incubation using Twist Core Exome probes according to manufacturer's protocol. Post-capture library pools were quantified by Qubit fluorometer and Agilent TapeStation 2200. Library pools were evaluated for quality and pool balance with shallow sequencing on an Illumina MiSeq, and were sequenced on an Illumina NovaSeq6000 100 bp read pairs by the Molecular Biology Core Facilities at Dana-Farber Cancer Institute. For samples MUV78, MUV35, MUV87, MUV16, MUV77, MUV17 and MUV86, whole-exome libraries were prepared using TruSeqExome Kit (Illumina) and sequenced on a NextSeq500 (150 cycles)[1].

Targeted exome-sequencing. For most samples profiled by targeted panel next-generation sequencing (NGS), the previously validated OncoPanel assay was performed at the Center for Cancer Genome Discovery at Dana-Farber Cancer Institute for 447 cancer-associated genes[2]. In brief, 50-200 ng tumor DNA was hybridized using a solution-phase Agilent SureSelect TM hybrid capture kit and sequenced using an Illumina HiSeq 2500 sequencer with 2x100 paired-end reads. Sample UMPED65 was profiled using the Illumina TruSight Oncology 500 (TSO500) NGS assay. Sample AAA010043 was profiled using the OncoKids™ Cancer Panel at CHLA[3].

Tumor tissue sectioning for RNA *In Situ* Hybridization, immunofluorescence, hybridization-based *in situ* sequencing (HybISS). Fresh tumor tissue was frozen immediately. After OCT embedding, 10 μm sections were sectioned and mounted on VWR superfrost Plus slides.

RNA *In Situ* Hybridization. Sections were stained using RNAscope 2.5 HD Duplex Detection Kit (Advanced Cell Diagnostics (ACD), 322430) according to manufacturer's instructions. Slides were fixed in 4% PFA for 15 min at 4 °C, followed by sequential dehydration in 50%, 70%, and 100% ethanol for 5 min each at RT. Tissue was treated with RNAscope Hydrogen Peroxide (ACD, 322335) for 10 min at RT, followed by RNAscope Protease IV (ACD, 322340) for 30 min at RT. ACD RNAscope probes used were Hs-CD44 (ACD, 311271), and Hs-CD14 (418801-C2). Hybridization probes were prepared by diluting C2 probe (red, alkaline phosphatase) 1:50 into C1 probe (green, horseradish peroxidase). Probes were hybridized for 2 h at 40 °C, followed by ten amplification steps according to RNAscope 2.5 HD Duplex Detection Kit protocol. Tissue was counterstained with Gill's Hematoxylin I (Statlab, HXGHE1LT) for 30 s at RT and 0.02% ammonia water for 15 s. Microscope images of stained tissue sections were taken on a DMi8 brightfield microscope (Leica Microsystems) and LAS-X software (Leica Microsystems). Images were processed using ImageJ (version 2.1.0/1.53C, RRID:SCR_003070).

Probe Design for HybISS. Padlock probes were designed for the selected genes, each containing two arms together matching a 40-base-pair (bp) sequence on the cDNA, a 4-bp barcode, an 'anchor sequence' allowing all amplicons to be labeled simultaneously, and a 20-bp hybridization sequence for additional readouts.

Target sequences for the selected genes were obtained using in-house Python padlock design software package that utilizes ClustalW and BLAST+ (https://github.com/Moldia/multi_padlock_design) with the following parameters: arm length, 15; Tm, low 65, high 75; space between targets, 15. After target sequences were obtained, five targets were selected randomly per gene. If fewer targets were found, then only those were selected. The backbone of the PLPs include a 20 nucleotide (nt) ID sequence and a 20 nt 'anchor' sequence that is common among subsets of PLPs (Note: in this study, the anchor sequences were not used and served only as linker sequences) (see Supplementary Table 6).

Mutation Primer Design for HybISS. LNA primers were designed against both *H3-3A* and *HIST1H3B* for mutant and wildtype targets, respectively[4]. The designs are as follows: H3F3Ac.83A>T (p.K28M) sequence +AG+TACCA+GGCCTG+TA+AC+GA+TGAGGT, and HIST1H3Bc.83A>T(p.K28M) sequence +AG+TGCCCGG+GCGG+TA+ACGGTG+AGGC+T. LNA base are indicated by a '+' sign (+G, +A, +T, +C). The primers were synthesized by Qiagen as custom LNA oligonucleotides 25 nmol. The LNA primers were added along with random decamers to increase signal amplification for mutant and WT targets during the reverse transcription step.

Imaging for HybISS. Imaging was performed using a standard epifluorescence microscope (Zeiss Axio Imager.Z2) connected to an external LED source (Lumencor® SPECTRA X light engine). Light engine was set up with filter paddles (395/25, 438/29, 470/24, 555/28, 635/22, 730/40). Images were obtained with a sCMOS camera (2048 × 2048, 16-bit, ORCAFlash4.0 LT Plus, Hamamatsu), automatic multi-slide stage (PILine, M-686K011), and Zeiss Plan-Apochromat

objectives 20X (0.8 NA, air, 420650-9901), 40X (1.4 NA, oil, 420762–9900). Filter cubes for wavelength separation included quad band Chroma 89402 (DAPI, Cy3, Cy5), quad band Chroma 89403 (Atto425, TexasRed, AlexaFluor750), and single band Zeiss 38HE (AlexaFluor488). Each field-of-view (FOV) was imaged with 21 z-stack planes with 0.5 µm spacing and 10% overlap between FOVs.

Immunofluorescence (IF) Staining. IF was performed on the same sections processed via HybISS after the final cycle was collected. After stripping the DIPG tissue sections of the HybISS detection oligonucleotides, the sections were washed twice with 2xSSC and once with PBS. For blocking, the samples were incubated in PBTA (PBS, 5% f.c. normal donkey serum, and 5% TBS Tween-20) for 1-2 hours. The samples were then washed twice with PBS. Primary antibodies were prepared to a final volume of 200 µl per slide in PBS as follows: recombinant Anti-Histone H3 mutated K27M (Abcam ab190631) 1:5,000, and 2% f.c. normal donkey serum. The tissue sections were incubated with primary antibody at 4 °C overnight and then washed twice with PBS. Secondary antibodies were prepared to a final volume of 500 µl per slide in PBS as follows: Goat Anti-Rabbit IgG H&L (Alexa Fluor 647) preadsorbed (Abcam ab150083) 1:4,000 and 0.5% f.c. DAPI. The sections were incubated at RT for 1-2 hours, followed by washing 3x with PBS. For imaging, the slides were mounted with SlowFade Gold Antifade Mountant (Thermo Fisher Scientific) and covered with glass coverslips.

Identification of copy number alterations in scRNA-seq data. Copy number alterations (CNAs) were estimated with inferCNV[5]. We sorted genes according to their chromosomal locations and calculated the mean relative expression of a sliding window of 100 genes per chromosome. To

determine the presence of CNA, we applied hierarchical clustering to the single cell copy number profiles within each sample with 190 (fresh samples) or 54 (frozen samples) copy number profiles from previously identified non-malignant cells[6,7]. For the majority of tumors (12/12 fresh, 21/25 frozen), most cells exhibited clear evidence of CNAs and did not cluster with the spike-in non-malignant cells.

Identification of non-malignant cell types. Malignant and non-malignant cells were clustered based on their transcriptional profiles and marker gene expressions were examined in each cell cluster. Data from scRNA-seq and snRNA-seq were analyzed separately to avoid technical artifacts. Briefly, we selected highly variable genes (HVGs) using Seurat's[8] FindVariableGenes and default parameters. We then used relative expressions of these HVGs for principle component analysis (PCA) and the top 16 (scRNA-seq) or 15 (snRNA-seq) principal components (PCs) for determining a UMAP embedding using Seurat's RunUMAP and default parameters. We clustered cells with the Louvain algorithm implemented by Seurat's FindClusters at a resolution of 1 (scRNA-seq) or 0.5 (snRNA-seq). Five clusters included cells from multiple patients. One of these clusters exhibited expression of cell-cycle related genes (*CDC20*, *CCND1*, *TOP2A*), indicating actively dividing cells. The other four clusters showed high expression of marker genes for non-malignant cell types, including microglia (*CD14*, *FCER1G*, *CSF1R*), oligodendrocytes (*MBP*, *PLP1*, *MOG*), T cells (*SKAP1, CD8A, CD247*), and endothelial cells (*CLDN5, IFITM1*, *ESAM*) (Extended Data Figure 1). These cells were also classified not to possess CNAs. For mere visualization in Figure 1c, we integrated scRNA-seq/snRNA-seq data using Harmony[9].

Integrated definition of malignant cells. We combined CNA classification with expression of non-malignant marker genes for malignancy classification. Non-malignant cells were grouped separately, showed no apparent CNAs, and expressed high levels of canonical marker genes. In fresh tumors, malignant cells did not cluster with non-malignant cells, and were classified to harbor CNAs. Fresh cells with discordant classifications by marker gene expressions and CNA classifications were excluded from downstream analysis. A group of cells from MUV17, that did not show any CNA and did not cluster with non-malignant cells, were determined as malignant based on detection of the H3-K27M mutation with targeted sequencing[6]. 21/25 frozen tumors were found to harbor cells with CNAs, while 4 tumors (SUDIPG55, MUV82, pSCG1, pTG5) did not present with an evident CNA signal. This could be either due to absence of CNAs in malignant cells in this sample or due to technical artifacts arising from snRNA-seq data. If these cells without evident CNAs did not cluster with the non-malignant spike-ins in subsequent clustering, they were retained as malignant cells. Following QC filtering and malignancy calling, 6 frozen tumors (SUDIPG30, SUDIPG31, SUDIPG52, pTG4, S1D2E3H7, MUV16) had < 30 malignant cells retained and were removed from tumor heterogeneity analyses.

Generation of scRNA-seq expression scores. Given a set of genes (*Gj*) (e.g., a metaprogram), a score, SC*j*(*i*), which quantifies the relative expression of *Gj* for each cell *i*, was computed as the average relative expression (Er) of the genes in *Gj*, compared to the average relative expression of a control gene set Gcont: SC (i) = average[Er(G , i)]−average[Er(Gcont, i)]. For each gene within the gene set, the control gene set contains 100 genes with the most similar aggregate expression level to that gene. Therefore, the control gene set represents a comparable distribution of expression levels to that of the considered gene set, and the control gene set is 100-fold larger.

Assignment of metaprograms to cells and identification of metaprogram-specific signature genes. Single cells were assigned to the metaprogram with the maximum expression score, and cells were classified as cycling, if scores of either S or G2M metaprograms were >1 in scRNA-seq, or >0.5 in snRNA-seq.

We identified signature genes for each cell population that was assigned to a metaprogram using Wilcoxon rank-sum test (log fold change >0.5 and minimally detected in 30% of cells assigned to a metaprogram). We kept genes that showed a Bonferroni-adjusted p-value <0.05 as metaprogram specific signature genes (Supplementary Table 2).

Characterization of metaprograms. Besides manually inspecting gene signatures, we characterized the metaprograms by four complementary approaches[7,10]. (1) We tested for enrichment of defined gene sets (Gene Ontology biological processes) in each metaprogram using a hypergeometric test. (2) We determined single-cell expression scores of non-malignant cell types for each malignant metaprogram. We collected scRNA-seq data for non-malignant brain cells from multiple human and mouse brain atlas datasets[11-13]. For each source, we aggregated cells by their reported cell type annotation, defined the mean expression profile of each cell type, and computed expression scores of each malignant metaprogram in all cell types as described above. (3) We identified the top 50 differentially expressed genes (ranked by Bonferroni adjusted p-value) of each reported cell type within the normal brain atlas using Seurat's FindAllMarkers function (Wilcox rank-sum test, log fold change >0.7 and minimally detected in 30% cells) as marker gene sets of each normal cell type. We then computed expression scores of normal gene sets in each malignant cell. (4) We characterized the expression similarities (Pearson correlations) between

mean expression profiles of normal cell types used in (2) and malignant cell types defined by NMF metaprograms. HVGs identified from both normal atlas datasets and this DMG malignant dataset (see earlier methods) were pooled and used for Pearson correlation calculation to minimize background noise (Figures 3d-f; Extended Data Figures 3a-c).

Pseudotime analysis of different OPC-like subpopulations. We inferred pseudotime ordering of all non-cycling OPC-like cells by Slingshot[14]. Slingshot constructs a cluster-based minimal spanning tree to identify global lineage structure and fits a principal curve to represent each lineage. We first computed a diffusion map embedding using log2 transformed expression levels of top 2,000 highly variable genes and default values for other parameters by the R package destiny's DiffusionMap function. The first two dimensions of the resulting diffusion map (DC1/2) were used for downstream analysis. Slingshot's slingshot function with default values for other parameters was used for pseudotime inference, without specifying a starting cell subpopulation. We next fit a general additive model (GAM) to each gene to identify temporally dynamic genes that change their expression over the course of the trajectory and we retained genes with adjusted p-values less than 0.05.

Gene regulatory and TF network reconstruction. To characterize underlying gene regulatory networks (GRNs) in our scRNA-seq dataset, the single-cell regulatory network inference and clustering (SCENIC) package was employed to identify gene regulatory modules with a *cis* regulatory binding motif for upstream regulators[15]. We extracted coexpression modules by GENIE3 that constructs regression models to predict expressions of target genes from the expression of TFs and uses importance of TFs in the regression model to determine significance

of regulatory interactions. We then applied RcisTarget to assess the enrichment of *cis* regulatory motif in target genes and prune indirect targets lacking motif binding site. We next estimated activities of the resulting regulatory modules (regulons) in single cells using AUCell. The regulon activities were Z-score normalized into relative activities and aggregated relative activity of each TF regulon was computed as mean relative activity of each cell subpopulation. To identify cell type specific TF regulons, we computed a regulon specificity score (RSS) by gauging the distance between the distribution of regulon activities and the distribution of cell type or metaprogram annotations using the Jensen-Shannon Divergence[16]. For each cell population, the RSS for all TF regulons was ranked from high to low and cell population specific regulons were selected as highest ranked outliers.

Ligand-receptor interactome analysis. We utilized CellChat to interrogate the cross-talk between different OPC-like populations and myeloid cells in the tumor microenvironment. CellChat relies on a manually curated signaling molecule interaction database (CellChatDB) and identifies significantly overexpressed ligands and receptors for each cell subpopulation[17]. CellChat next quantifies interaction probability by the law of mass action based on the average expression values of ligands and receptors in sender and receiver cell subpopulations, respectively, and assesses statistical significance by a permutation test that randomly shuffles group labels of cells. We applied CellChat to each sample separately and only focused on genes that were detected in 30% of at least one cell subpopulation with log fold change >0.5. The resulting ligand-receptor interactions were filtered by p-values and the number of samples significant for these interactions were identified; only those with adjusted p-values <0.05 in at least three samples were retained.

Identification of CNAs and integrated definition of malignant nuclei profiled by snATAC-seq. Copy number alterations (CNAs) were estimated by running inferCNV on estimates of gene activities, similarly to determination of CNA in scRNA-seq data[5]. We then applied hierarchical clustering to single-nucleus copy number profiles of each sample with those of putative normal cells to determine whether a nucleus encompasses CNAs or not. In most samples with both scRNA- and snATAC-seq data, similar CNAs were identified in both modalities.

We combined CNA classification with chromatin accessibility of non-malignant marker genes to identify malignant and non-malignant nuclei. Non-malignant nuclei showed no apparent CNAs and high activities of canonical non-malignant marker genes. In contrast, malignant nuclei did not cluster with non-malignant nuclei, and were classified to harbor CNAs. Nuclei with discordant classifications by marker gene expressions and CNA classifications were excluded from downstream analysis.

Clustering of malignant nuclei in snATAC-seq. After removing non-malignant nuclei, we focused on analysis of malignant nuclei alone using Signac. Briefly, we normalized the data using RunTFIDF and conducted dimensionality reduction using RunSVD and the top 25% of features. To integrate multiple samples, we applied a linear adjustment to the resulting 2-50 LSI components with Harmony, omitting the first LSI component as it showed a strong correlation with sequencing depth. We then calculated k-nearest neighbours using FindNeighbours and top 30 Harmony components. We identified cell clusters by SNN clustering algorithm and running FindClusters function (algorithm=3/SLM and resolution=0.6) and generated a UMAP embedding using RunUMAP function with top 30 Harmony components. Due to sparsity of snATAC-seq data and

highly variable CNAs in different samples, different samples were only partially integrated (Extended Data Figure 4c).

To minimize impact of sample-specific variation from variable CNAs, we also analyzed each sample separately with the same data normalization (RunTFIDF), dimension reduction (RunSVD), KNN construction (FindNeighbours), SNN clustering (FindClusters), and UMAP generation (RunUMAP) methods. For KNN clustering, 2-30 LSI components and SLM algorithm were used, with resolution set to 0.6. Gene activity and DAGs were computed similarly as described in a previous section. Top DAGs were utilized to annotate each cell cluster. OPC-like (e.g., *MYT1, EPN2, CSPG4*) and AC-like (e.g., *HSP8, AQP4*) nuclei were identified in all samples. OC-like (e.g., *BCAS1, SOX10, MBP*) nuclei were identified in MUV35 and MES-like (e.g., *GAP43, CHI3L1, ANXA2*) nuclei were identified in MUV82 and MUV86. AC-like-alternative nuclei with enhanced accessibility for synaptic marker genes (e.g., *GABBR2, GRIA1, CAMK2B;* see also below) were identified in MUV1 (Extended Data Figure 4d). Of note, and unlike in our scRNA-seq analysis, we did not detect nuclei to form a distinct 'cycling' cluster based on accessible chromatin profiles alone. This has been previously reported and may be attributed to the preferential recapitulation of cell lineages instead of more transient cell states at the chromatin level[18,19].

We combined annotations of each sample as the joint annotation of all samples and mapped them onto the joint UMAP (Figure 4a). We identified median numbers of 5,268 accessible chromatin sites in OPC-like cells, 5,389 in OC-like cells, 4,065 in AC-like cells, and 4,861 in MES-like cells. We next identified DAGs similarly as described in a previous section amongst all nuclei.

We then identified differentially accessible peaks (DAPs) amongst all nuclei. Peaks that were identified in at least 20% of nuclei within each malignant cell type were tested by Wilcoxon rank-

sum test with Bonferroni multiple test correction, and peaks with log fold change >0.1 and adjusted p-value <0.05 were selected. DAPs were linked to their nearest genes by the ClosestFeature function.

Annotation of the AC-like-alternative cluster

The only tumor cell subpopulation that was not detected by scRNA-seq, but snATAC-seq only, was the 'AC-like-alternative' cell group that depicted enhanced chromatin accessibility for a synaptic signature (Figures 4a-b; Extended Data Figures 4d-f). Previous work has identified a subgroup of malignant cells in H3-K27M DMGs enriched for synaptic gene expression that engages in functional neuron-to-glioma synapses[20]. This synapse-associated gene expression was found chiefly in OPC-like glioma cells[20] in a scRNA-seq dataset gleaned from younger children with H3-K27M DMG[6]. Here, the synaptic signature-expressing cells clustered closely to AC-like cells by their open chromatin profiles, scored highly for AC-like marker gene activities (e.g., *AQP4, SPARC*), and were mainly annotated as 'AC-like' cells upon cross-modality integration with scRNA-seq profiles (Figures 4a-b; Extended Data Figure 4g). Furthermore, this population depicted low chromatin accessibility for canonical neuronal lineage and OPC-like marker genes (Extended Data Figure 4e). These observations indicate that this synaptic population presents a rare subpopulation of AC-like cells that is enriched for genes associated with synaptic function and that is distinct from the previously described more frequent synaptogenic OPC-like subpopulations in H3-K27M DMG enriched for different glutamatergic genes[20]. The capacity of astrocyte(-like) subpopulations in expressing synapse-associated signatures and supporting synapse formation has been described in the normal brain and in glioblastoma[21]. Together, this data raises the possibility that both OPC-like and AC-like malignant cells may be engaged in

neuron-glioma interactions in H3-K27M DMG, which needs to be validated in future functional studies.

Processing and variant calling from WES data. Raw sequencing reads were aligned to hg19 reference genome using Burrows Wheeler aligner BWA-MEM v0.7.1522. Read duplicates were removed from bam files using MarkDuplicates (samtools v1.3.1), followed by base recalibration using BaseRecalibrator (GATK 4.1.9.0) and ApplyBQSR from GATK. Germline mutations for BaseRecalibrator and all other steps requiring germline mutation info were downloaded from GATK best practices, b37, gnomad vcf (https://console.cloud.google.com/storage/browser/_details/gatk-best-practices/somatic-b37/af-only-gnomad.raw.sites.vcf;tab=live_object). Panel of normals (PoN) were either 1) downloaded from GATK best practices, B37 WES PoN (https://console.cloud.google.com/storage/browser/_details/gatk-best-practices/somatic-b37/Mutect2-exome-panel.vcf;tab=live_object), or 2) derived from sequencing 13 non-tumor tissue areas of autopsy brains (DIPG17_normal, DIPG24_normal, DIPG29_normal, DIPG31_normal, DIPG33_normal, DIPG36_normal, DIPG38_normal, DIPG39_normal, DIPG45_normal, DIPG52_normal, DIPG67_normal, pTG4_normal, pSCG1_normal; all deposited in EGA (EGAS00001006431)). Variant calling was performed using mutect2 (GATK 4.1.9.0) with one of the following settings: 1) tumor only mode, using GATK PoN; 2) tumor only mode, using PoN created from 13 normal samples; 3) paired tumor/normal mode, for the 5 samples with both tumor and normal data available, using the GATK PoN. Prior to variant filtration, contamination was calculated using GetPileupSummaries and CalculateContamination from GATK. The germline mutation file used for BaseRecalibrator was also used for both interval and

variant files for GetPileupSummaries. Variants were filtered with FilterMutectCalls from GATK, using the contamination tables generated by CalculateContamination. Variants were then annotated using Funcotator from GATK, with flag –remove-filtered-variants. Any filtered variants present in the PoN used for variant calling were filtered out. Samples MUV78, MUV35, MUV87, MUV16, MUV77, MUV17 and MUV86 were analyzed as described in Gojo et al. 2019[1].

Analysis of targeted exome-sequencing. For samples profiled by the DFCI OncoPanel assay, sequence reads were aligned to reference b37 edition from the Human Genome Reference Consortium using bwa and processed using Picard and GATK. Single-nucleotide variants (SNVs) were called using MuTect v1.1.4, and insertions and deletions (INDEL) were called with GATK Indelocator. For UMPED65, which was profiled using TSO500 assay, sequence reads were aligned to hg19 genome. Variant calling was performed using the Pisces software[23], and local INDEL realignment, paired-end stitching and read filtering to further improve variant calling results were carried out by the Gemini software[24]. Sample AAA010043 was mapped to hg19 genome, SNVs and INDELs were annotated using a customized variant curation tool[3].

Assessment of pciSeq marker gene panel. We validated the robustness of the marker gene panel used for pciSeq by comparing cell state classifications from all the curated 116 genes to classifications based on the single best marker gene for each cell state that in scRNA-seq is almost exclusively expressed in the respective malignant cell state only (*PDGFRA* for OPC-like, *RGR* for OC-like, *GFAP* for AC-like, *IGFBP3* for MES-like). We observed a high correlation between both gene sets (Pearson correlation: 0.83, Extended Data Figure 6c), which shows that the curated panel

of 116 marker genes is highly consistent with the top cell state specific marker genes to guide cell state classification.

For selection of marker genes for multiplexed IF, we selected single malignant cell state specific markers that our pciSeq analysis indicated to have high accuracy in cell state prediction when compared to probabilistic cell typing based on all 116 marker genes (Supplementary Fig. 6f) (Pearson correlation: 0.78). These markers showed both co-localization with anti-H3.3K27M staining as well as mutual exclusivity in IF, highlighting their validity as single markers specific for H3-K27M tumor cell populations (Fig. 6e; Supplementary Figs. 6g-h).

Supplementary Figures

Supplementary Figure 1

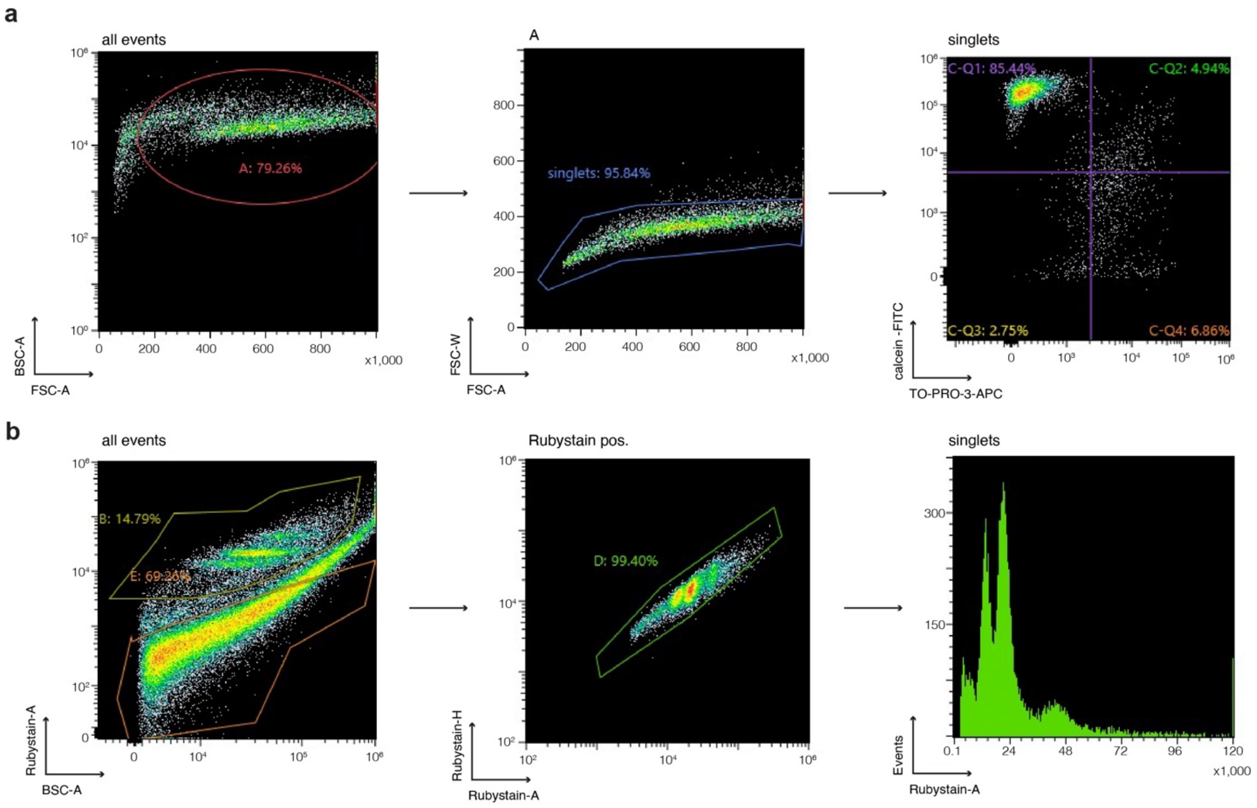

Supplementary Figure 1. Exemplary fluorescence-activated cell sorting (FACS) gating strategies. (a) for fresh single cells, (b) for frozen single nuclei.

Supplementary Figure 2

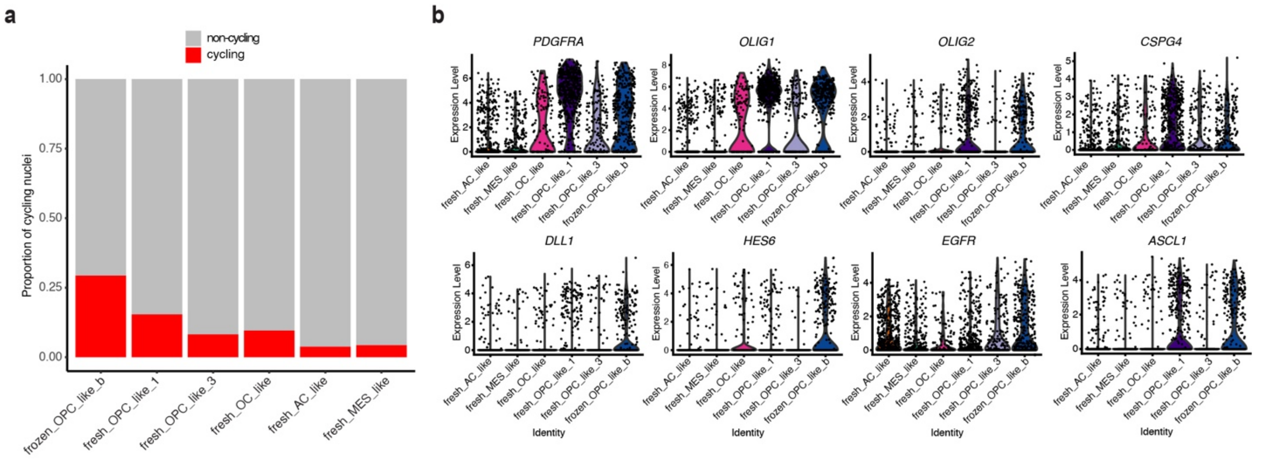

Supplementary Figure 2. OPC-like subpopulations in frozen snRNA-seq data. (a) Barplot showing frozen nuclei proportions (y-axis) annotated as cycling or non-cycling (color legend) across all projected fresh metaprograms and frozen OPC-like-b. (b) Violin plot representations of log normalized absolute expressions (y-axis) of canonical OPC and pre-OPC marker genes in all frozen nuclei across frozen OPC-like-b and fresh projected metaprograms (x-axis).

Supplementary Figure 3

AAA07653

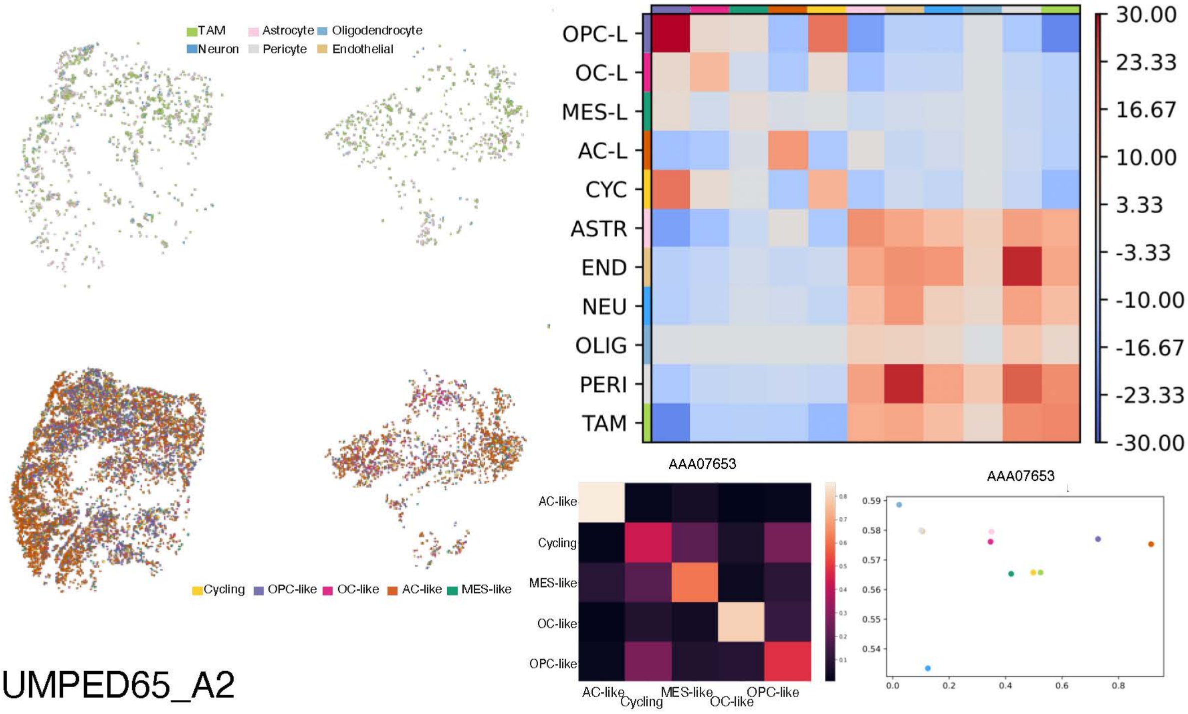

UMPED65_A2

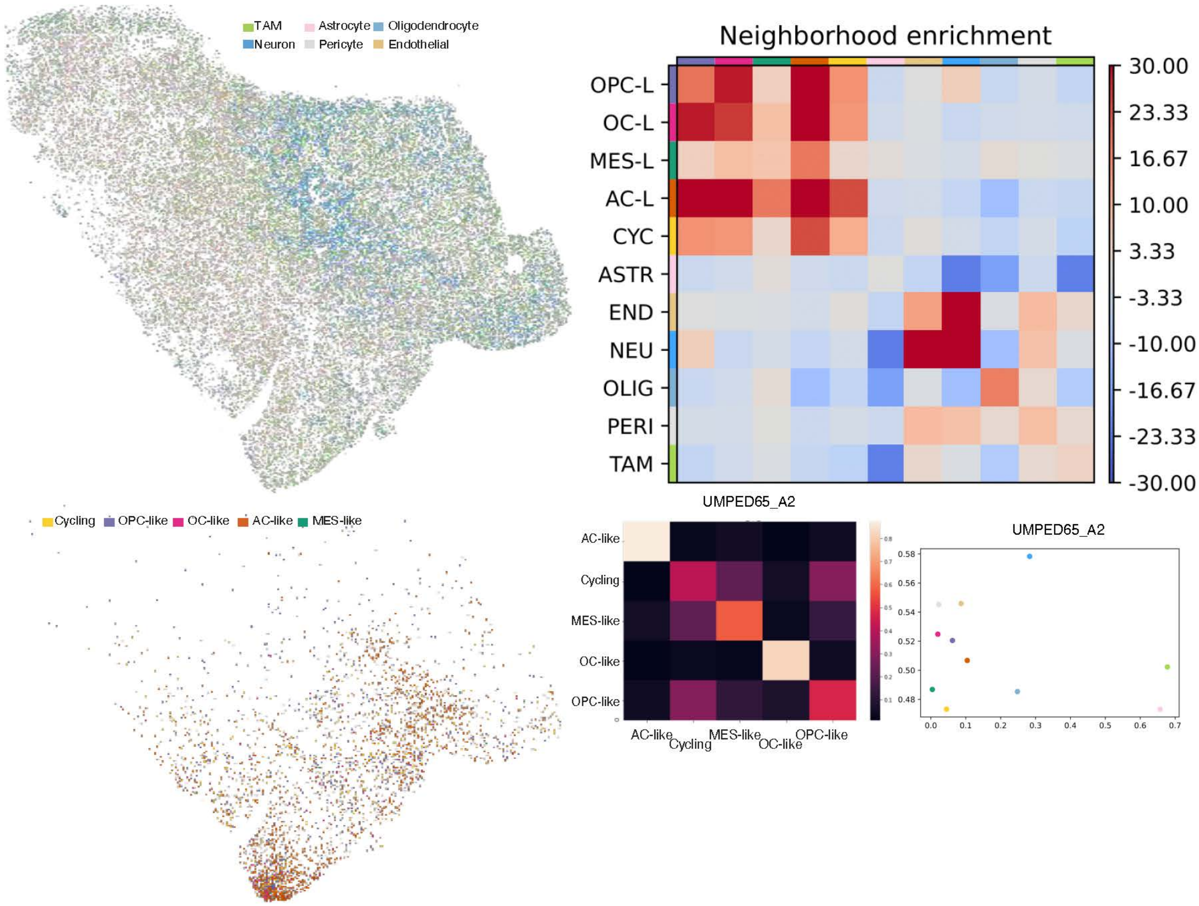

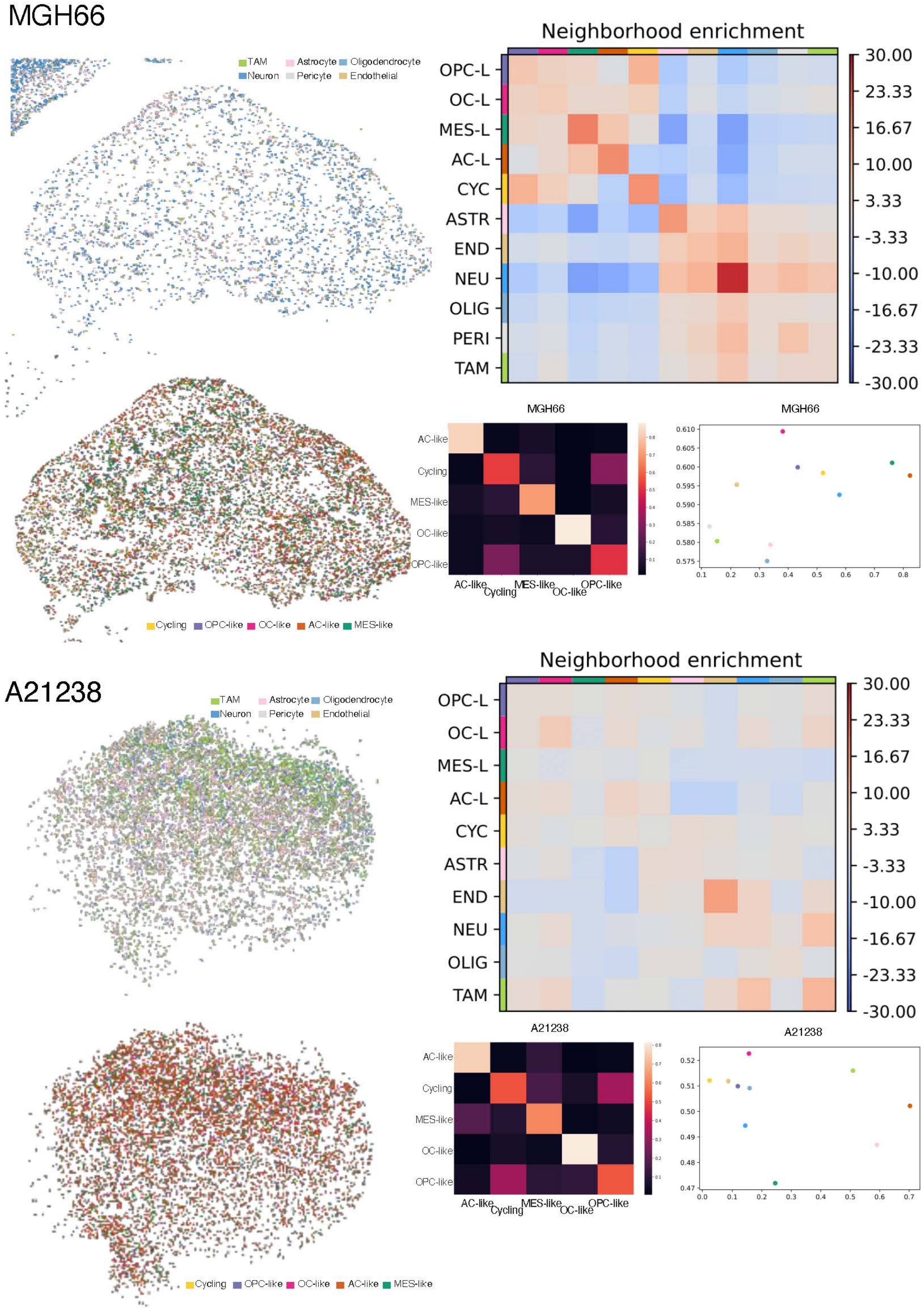
MGH66
Neighborhood enrichment
TAM
Astrocyte
Oligodendrocyte
Neuron
Pericyte
Endothelial
OPC-L
OC-L
MES-L
AC-L
CYC
ASTR
END
NEU
OLIG
PERI
TAM
30.00
23.33
16.67
10.00
3.33
-3.33
-10.00
-16.67
-23.33
-30.00
MGH66
AC-like
Cycling
MES-like
OC-like
OPC-like
MGH66
Cycling
OPC-like
OC-like
AC-like
MES-like
A21238
A21238
A21238

UMPED103_SVZ

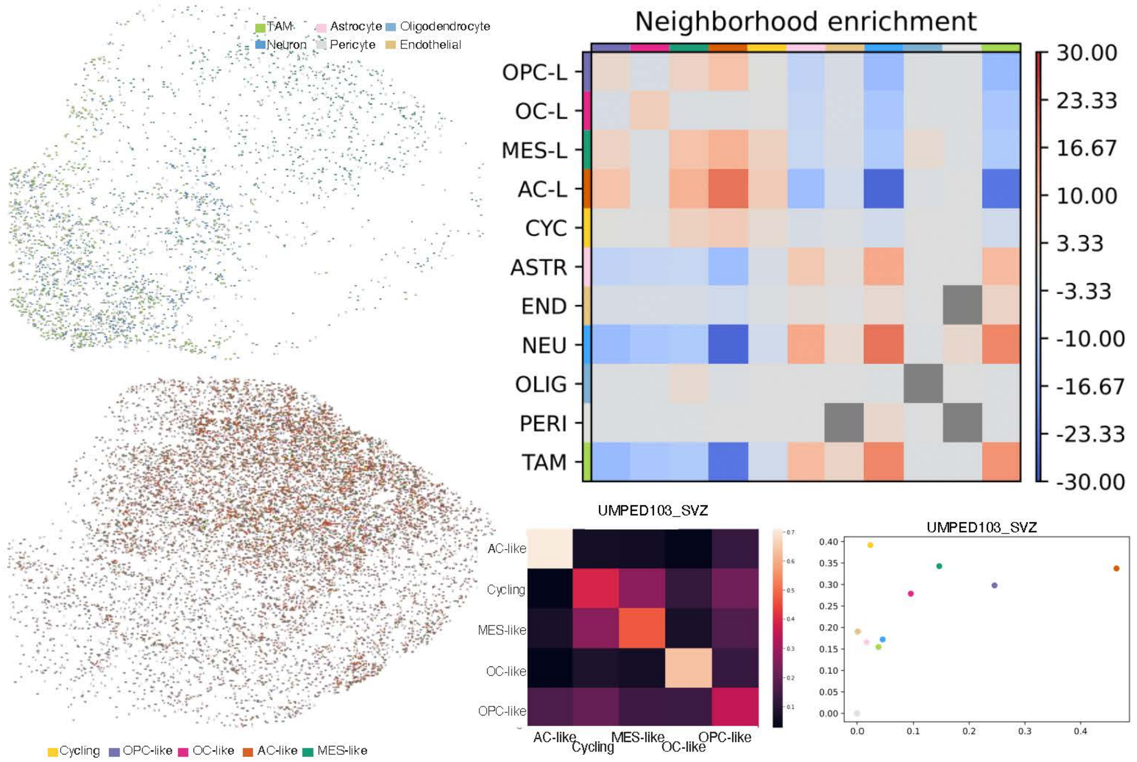

UMPED103_pons

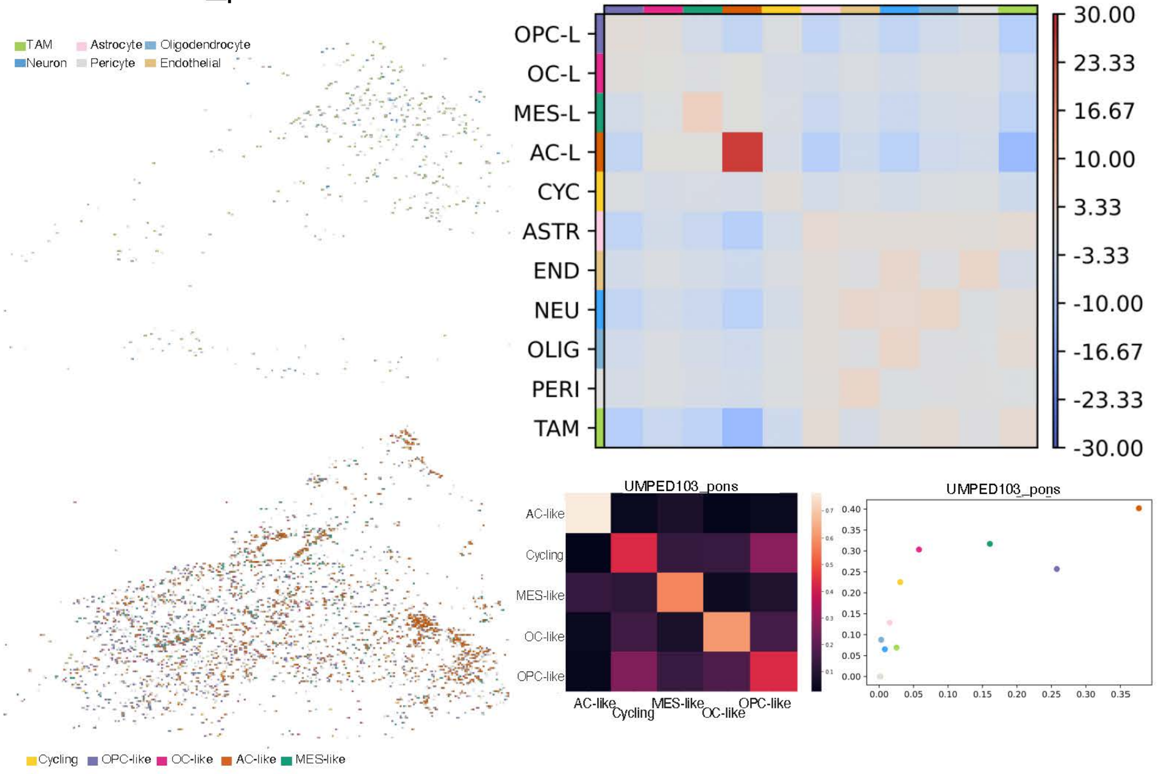

BT1873_pons

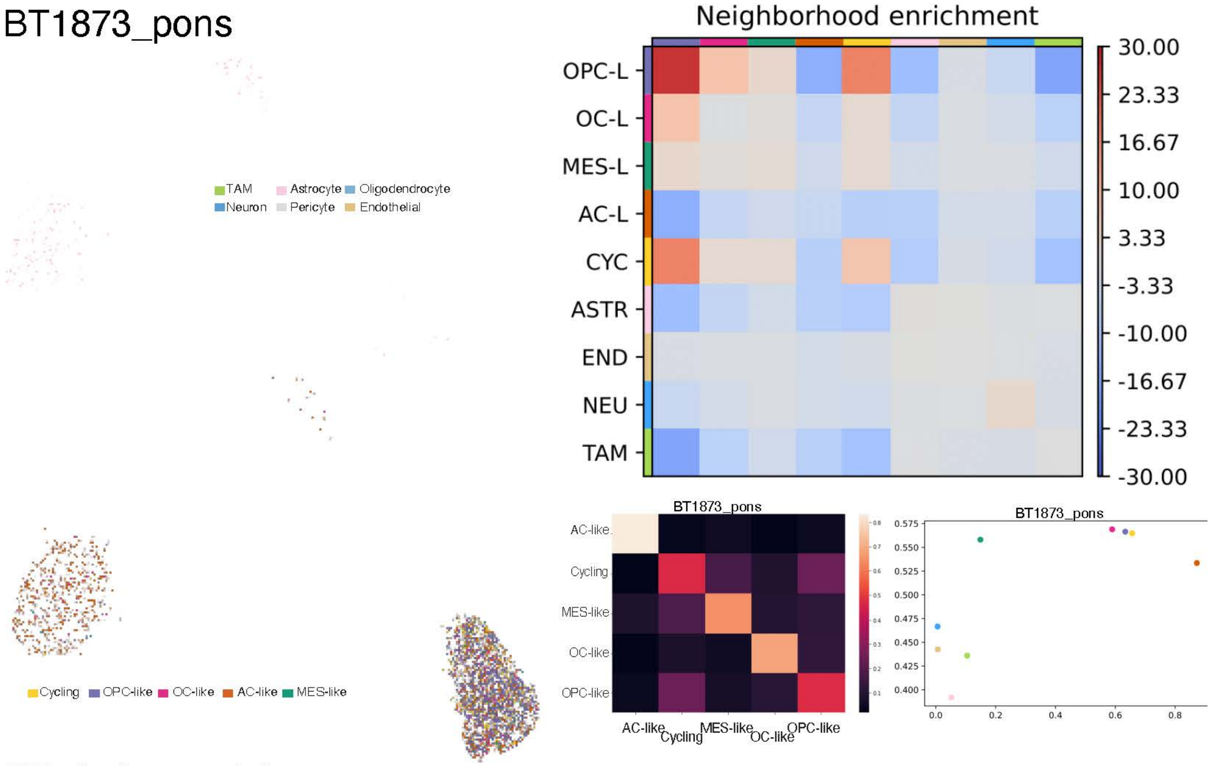

BT1873_ventricle

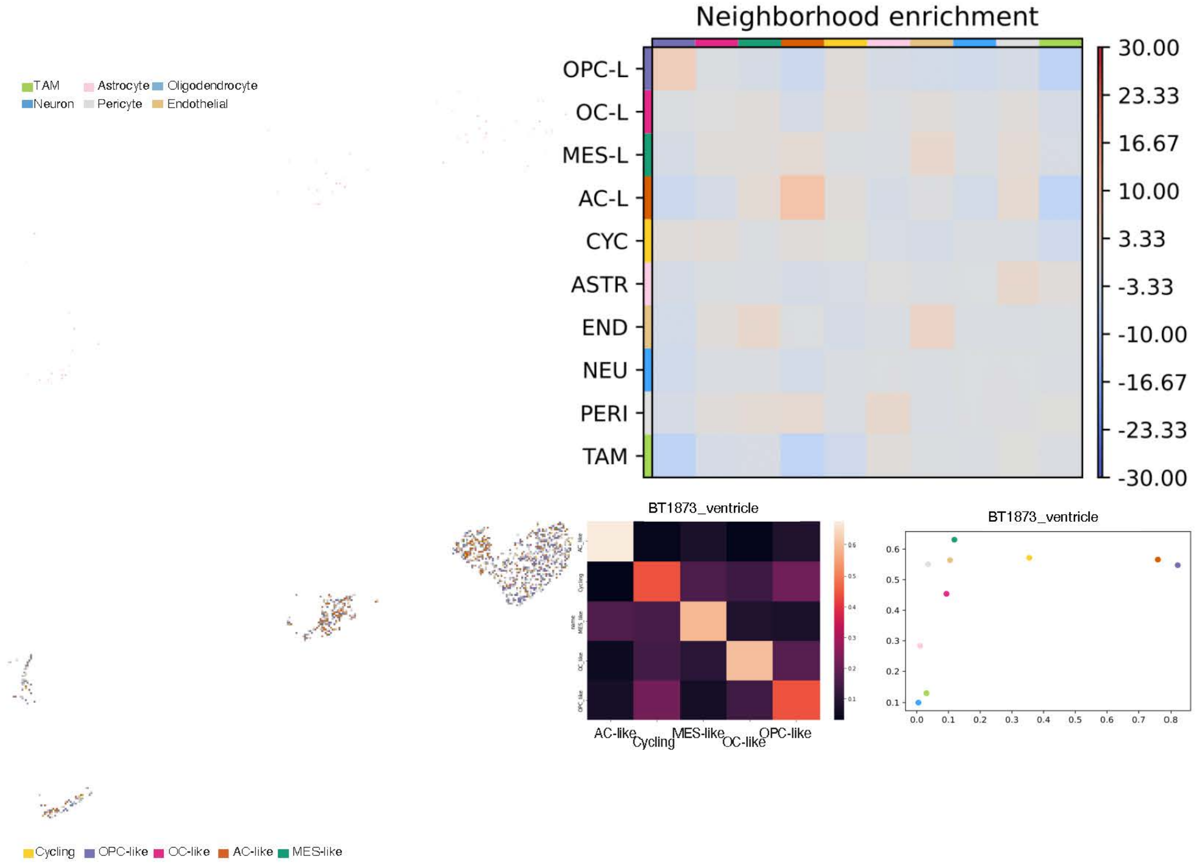

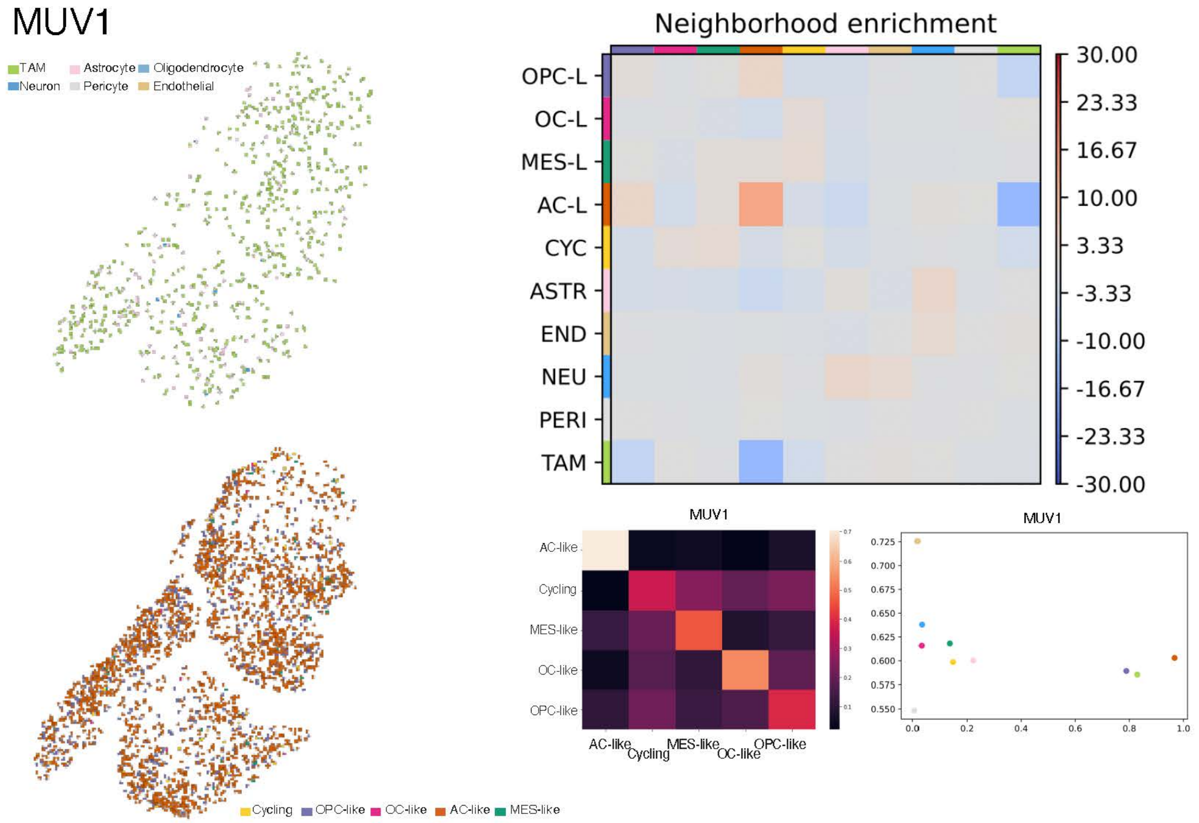
MUV1
TAM
Astrocyte
Oligodendrocyte
Neuron
Pericyte
Endothelial
Neighborhood enrichment
OPC-L
OC-L
MES-L
AC-L
CYC
ASTR
END
NEU
PERI
TAM
30.00
23.33
16.67
10.00
3.33
-3.33
-10.00
-16.67
-23.33
-30.00
MUV1
AC-like
Cycling
MES-like
OC-like
OPC-like
MUV1
Cycling
OPC-like
OC-like
AC-like
MES-like

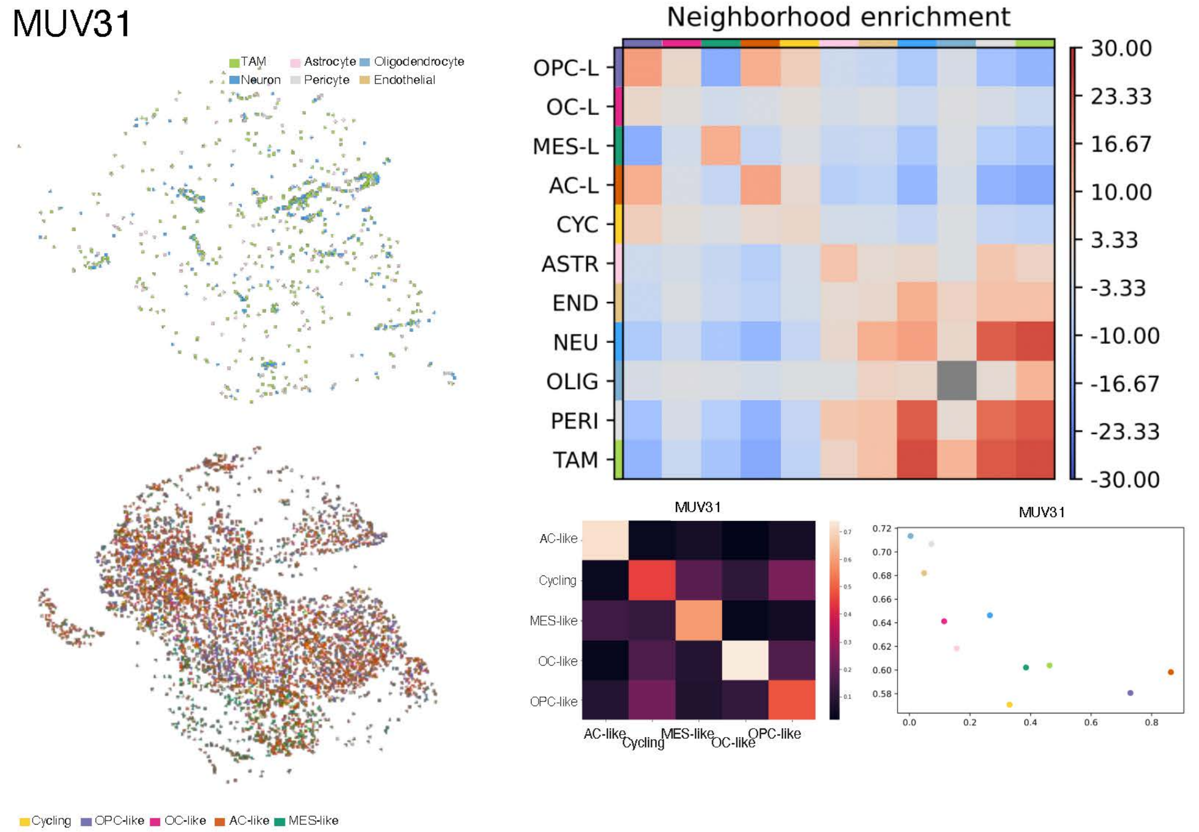
MUV31
TAM
Astrocyte
Oligodendrocyte
Neuron
Pericyte
Endothelial
Neighborhood enrichment
OPC-L
OC-L
MES-L
AC-L
CYC
ASTR
END
NEU
OLIG
PERI
TAM
30.00
23.33
16.67
10.00
3.33
-3.33
-10.00
-16.67
-23.33
-30.00
MUV31
AC-like
Cycling
MES-like
OC-like
OPC-like
MUV31
Cycling
OPC-like
OC-like
AC-like
MES-like

AAA010043

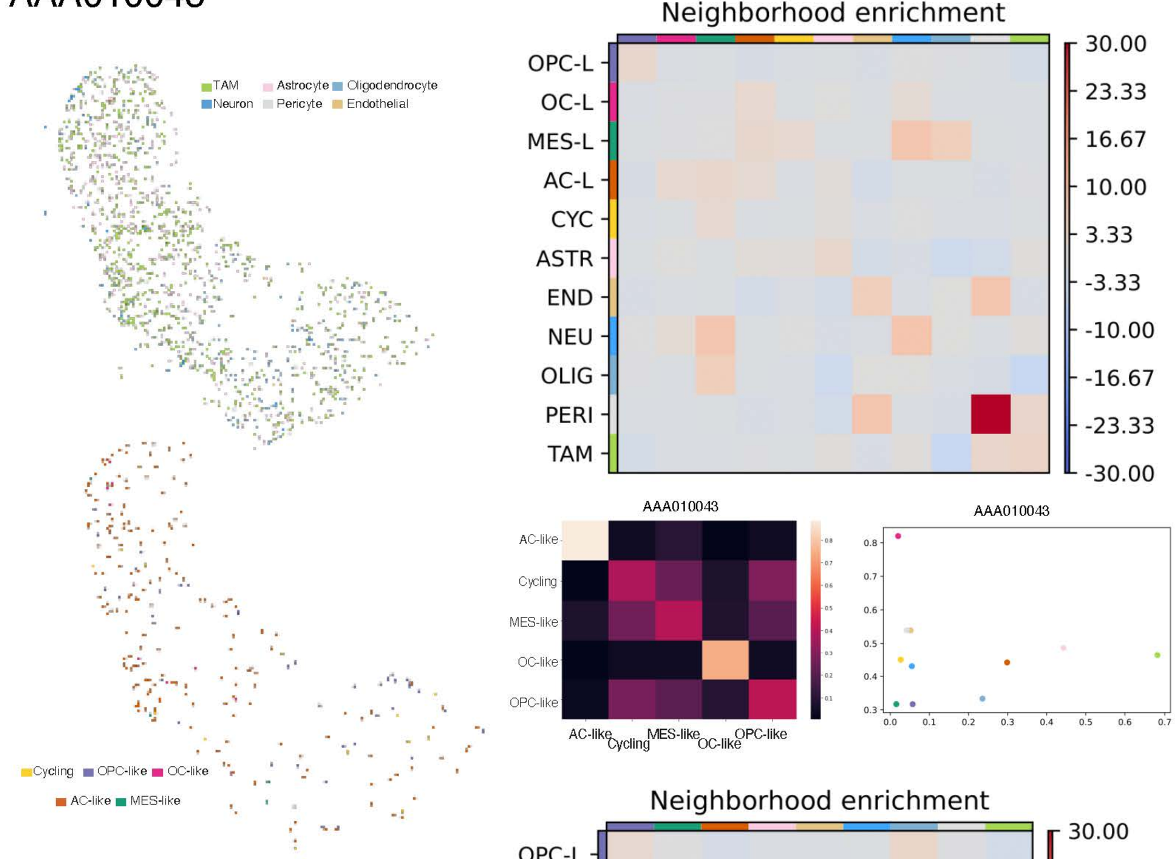

XT8698

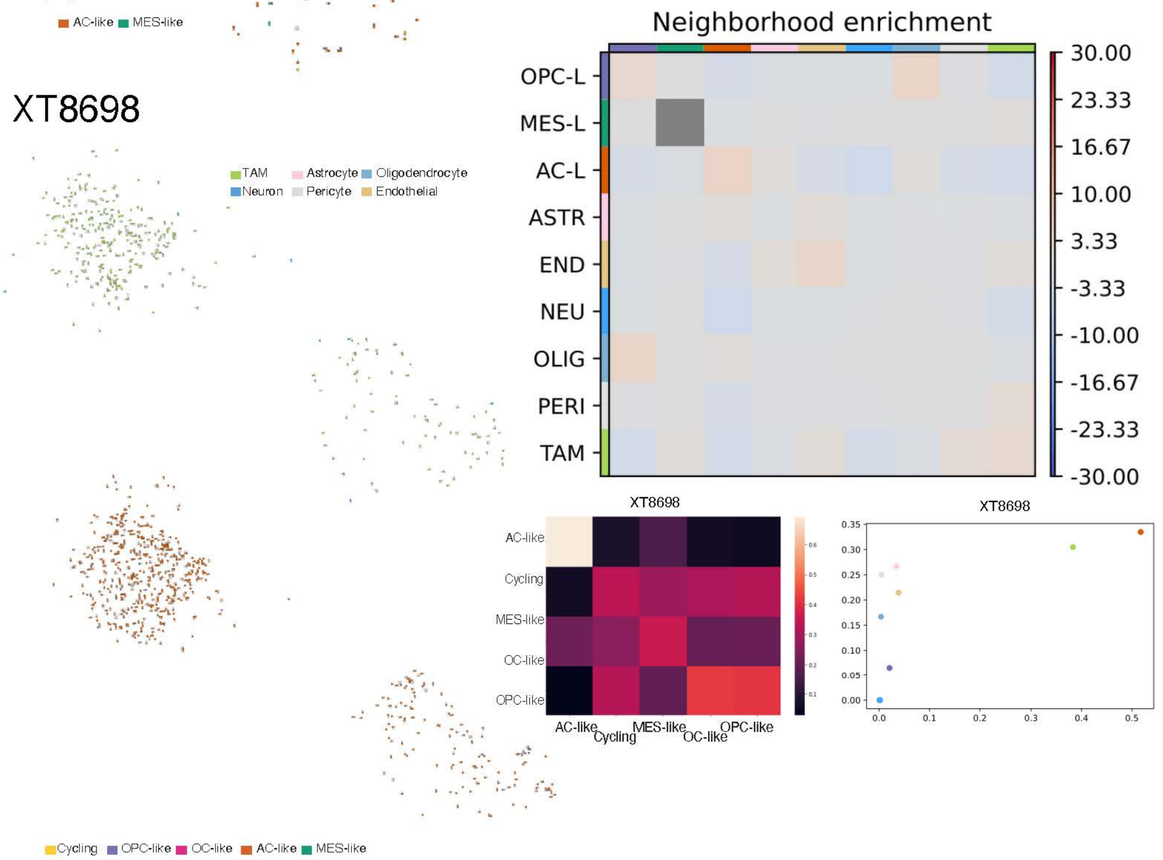

Supplementary Figure 3. Individual malignant and non-malignant cell state/type assignments, confusion matrices, neighborhood enrichment analyses, degree of centrality versus clustering coefficient scatter plots for each sample profiled by ISS, in which >1,000 high quality cells were analyzed: AAA07653, UMPED65_A2, MGH66, A21238, UMPED103_SVZ, UMPED103_pons, BT1873_pons, BT1873_ventricle, MUV1, MUV31, AAA010043, XT8698.

References supplementary information

1. Gojo, J. *et al.* Personalized Treatment of H3K27M-Mutant Pediatric Diffuse Gliomas Provides Improved Therapeutic Opportunities. *Front Oncol* **9**, 1436 (2019).
2. Ramkissoon, S.H. *et al.* Clinical targeted exome-based sequencing in combination with genome-wide copy number profiling: precision medicine analysis of 203 pediatric brain tumors. *Neuro Oncol* **19**, 986-996 (2017).
3. Hiemenz, M.C. *et al.* OncoKids: A Comprehensive Next-Generation Sequencing Panel for Pediatric Malignancies. *J Mol Diagn* **20**, 765-776 (2018).
4. Larsson, C., Grundberg, I., Soderberg, O. & Nilsson, M. In situ detection and genotyping of individual mRNA molecules. *Nat Methods* **7**, 395-7 (2010).
5. Tirosh, I. *et al.* Single-cell RNA-seq supports a developmental hierarchy in human oligodendroglioma. *Nature* **539**, 309-313 (2016).
6. Filbin, M.G. *et al.* Developmental and oncogenic programs in H3K27M gliomas dissected by single-cell RNA-seq. *Science* **360**, 331-335 (2018).
7. Gojo, J. *et al.* Single-Cell RNA-Seq Reveals Cellular Hierarchies and Impaired Developmental Trajectories in Pediatric Ependymoma. *Cancer Cell* **38**, 44-59 e9 (2020).
8. Stuart, T. *et al.* Comprehensive Integration of Single-Cell Data. *Cell* **177**, 1888-1902 e21 (2019).
9. Korsunsky, I. *et al.* Fast, sensitive and accurate integration of single-cell data with Harmony. *Nat Methods* **16**, 1289-1296 (2019).
10. Neftel, C. *et al.* An Integrative Model of Cellular States, Plasticity, and Genetics for Glioblastoma. *Cell* **178**, 835-849 e21 (2019).
11. Weng, Q. *et al.* Single-Cell Transcriptomics Uncovers Glial Progenitor Diversity and Cell Fate Determinants during Development and Gliomagenesis. *Cell Stem Cell* **24**, 707-723 e8 (2019).
12. Zhong, S. *et al.* Decoding the development of the human hippocampus. *Nature* **577**, 531-536 (2020).
13. Fu, Y. *et al.* Heterogeneity of glial progenitor cells during the neurogenesis-to-gliogenesis switch in the developing human cerebral cortex. *Cell Rep* **34**, 108788 (2021).
14. Street, K. *et al.* Slingshot: cell lineage and pseudotime inference for single-cell transcriptomics. *BMC Genomics* **19**, 477 (2018).
15. Aibar, S. *et al.* SCENIC: single-cell regulatory network inference and clustering. *Nat Methods* **14**, 1083-1086 (2017).

16. Van de Sande, B. *et al.* A scalable SCENIC workflow for single-cell gene regulatory network analysis. *Nat Protoc* **15**, 2247-2276 (2020).
17. Jin, S. *et al.* Inference and analysis of cell-cell communication using CellChat. *Nat Commun* **12**, 1088 (2021).
18. Ma, S. *et al.* Chromatin Potential Identified by Shared Single-Cell Profiling of RNA and Chromatin. *Cell* **183**, 1103-1116 e20 (2020).
19. Trevino, A.E. *et al.* Chromatin and gene-regulatory dynamics of the developing human cerebral cortex at single-cell resolution. *Cell* **184**, 5053-5069 e23 (2021).
20. Venkatesh, H.S. *et al.* Electrical and synaptic integration of glioma into neural circuits. *Nature* **573**, 539-545 (2019).
21. John Lin, C.C. *et al.* Identification of diverse astrocyte populations and their malignant analogs. *Nat Neurosci* **20**, 396-405 (2017).
22. Li, H. & Durbin, R. Fast and accurate short read alignment with Burrows-Wheeler transform. *Bioinformatics* **25**, 1754-60 (2009).
23. Dunn, T. *et al.* Pisces: an accurate and versatile variant caller for somatic and germline next-generation sequencing data. *Bioinformatics* **35**, 1579-1581 (2019).
24. Paila, U., Chapman, B.A., Kirchner, R. & Quinlan, A.R. GEMINI: integrative exploration of genetic variation and genome annotations. *PLoS Comput Biol* **9**, e1003153 (2013).
