# Supplementary material for: The landscape of tumor cell states and spatial organization in H3-K27M mutant diffuse midline glioma across age and location
Source: Nat Genet. 2022 Dec 5;54(12):1881–94. doi: 10.1038/s41588-022-01236-3 (PMC9729116; doi:10.1038/s41588-022-01236-3)
Supplement: Supplementary file 2 — Reporting Summary [file 41588_2022_1236_MOESM2_ESM.pdf]

nature portfolio

Corresponding author(s): Ilon Liu, Mariella G. Filbin

Last updated by author(s): 09/29/2022

Reporting Summary

Nature Portfolio wishes to improve the reproducibility of the work that we publish. This form provides structure for consistency and transparency in reporting. For further information on Nature Portfolio policies, see our Editorial Policies and the Editorial Policy Checklist.

Statistics

For all statistical analyses, confirm that the following items are present in the figure legend, table legend, main text, or Methods section.

n/a	Confirmed	
☐	☒	The exact sample size (*n*) for each experimental group/condition, given as a discrete number and unit of measurement
☐	☒	A statement on whether measurements were taken from distinct samples or whether the same sample was measured repeatedly
☐	☒	The statistical test(s) used AND whether they are one- or two-sided<br>*Only common tests should be described solely by name; describe more complex techniques in the Methods section.*
☐	☒	A description of all covariates tested
☐	☒	A description of any assumptions or corrections, such as tests of normality and adjustment for multiple comparisons
☐	☒	A full description of the statistical parameters including central tendency (e.g. means) or other basic estimates (e.g. regression coefficient) AND variation (e.g. standard deviation) or associated estimates of uncertainty (e.g. confidence intervals)
☐	☒	For null hypothesis testing, the test statistic (e.g. F, t, r) with confidence intervals, effect sizes, degrees of freedom and P value noted<br>*Give P values as exact values whenever suitable.*
☐	☒	For Bayesian analysis, information on the choice of priors and Markov chain Monte Carlo settings
☒	☐	For hierarchical and complex designs, identification of the appropriate level for tests and full reporting of outcomes
☐	☒	Estimates of effect sizes (e.g. Cohen's *d*, Pearson's *r*), indicating how they were calculated

Our web collection on statistics for biologists contains articles on many of the points above.

Software and code

Policy information about availability of computer code

Data collection	Single cell RNA-sequencing and ATAC-sequencing data was generated using the Illumina NextSeq 500 system according to the manufacturer's instructions. In situ sequencing images were acquired with a Zeiss Axio Imager.Z2 epifluorescence microscope. CODEX images were acquired on a Keyence BZ-X800E fluorescent microscope equipped with a BZ Nikon Objective Lens.
Data analysis	hisat2 v2.1.0<br>rsem v1.3.0<br>bwa v0.7.15<br>star v2.7.3a<br>samtools v1.3.1<br>chromHMM v1.22<br>GATK v4.1.9.0<br>HOMER v4.9.1<br>R v4.0.2<br>Python v3.9.6<br>ComplexHeatmap v2.4.3<br>ggplot v2 3.3.2<br>ggrepel v0.8.2<br>ggvenn v0.1.8<br>cowplot v1.1.0<br>inferCNV v1.4.0<br>weights v1.0.1<br>Seurat v2.3.4 and v3.2.2<br>Harmony v1.0<br>fgsea v1.14.0

clusterProfiler v3.16.1
NMF version 0.23.0
PAGODA2 v0.1.4
pheatmap v1.0.12
scCODA v0.1.4
SCENIC v1.2.2
GENIE3 v1.10.0
AUCell v1.12.0
GenomeInfoDb v1.26.1
EnsDb.Hsapiens.v75 v2.99.0
org.Hs.eg.db v3.12.0
GenomeInfoDb v1.26.1
GenomicRanges v1.42.0
Cell Ranger ATAC v1.0.1
Signac v1.1.0
FNN v1.1.3
ImageJ v2.1.0
pciSeq v0.0.45
Squidpy 1.1.2
Starfish 0.2.1
slingshot v1.99.14
cellchat v1.0.0
destiny v3.4.0
Custom scripts and code used in data processing and figure creation are available under: https://doi.org/10.5281/zenodo.7073167.

For manuscripts utilizing custom algorithms or software that are central to the research but not yet described in published literature, software must be made available to editors and reviewers. We strongly encourage code deposition in a community repository (e.g. GitHub). See the Nature Portfolio guidelines for submitting code & software for further information.

Data

Policy information about availability of data

All manuscripts must include a data availability statement. This statement should provide the following information, where applicable:

- Accession codes, unique identifiers, or web links for publicly available datasets
- A description of any restrictions on data availability
- For clinical datasets or third party data, please ensure that the statement adheres to our policy

ScRNA-seq and scATAC-seq data of primary patient DMGs have been submitted to GEO (GSE184357). ISS data are available at Zenodo under ID 6805729. Previously published scRNA-seq data reanalyzed in this study are available under accession codes GSE10213021, GSE12287142, GSE14446240, GSE13125843, and GSE12303051. WES data generated in this study is deposited in EGA (EGAS00001006431). For targeted exome-sequencing data, the majority of which was generated as part of routine clinical care, variant data has been included as Supplementary Table 7 for all samples except for A21-238 and AAA010043 as these were generated by external care providers with restricted data access. Previously published WGS data of tumors ICGC-GBM27, ICGC-GBM96, ICGC-GBM60 is deposited at EGA00001001139, and WGS data for BT836 and BT869 has been published under dbGaP accession number phs002380.v1.p183. H3-K27M DMG ChIP-seq data were utilized from GSE12631919.

Field-specific reporting

Please select the one below that is the best fit for your research. If you are not sure, read the appropriate sections before making your selection.

☒ Life sciences ☐ Behavioural & social sciences ☐ Ecological, evolutionary & environmental sciences

For a reference copy of the document with all sections, see nature.com/documents/nr-reporting-summary-flat.pdf

Life sciences study design

All studies must disclose on these points even when the disclosure is negative.

Sample size	Sample size was determined by the availability of donor and patient-derived material.
Data exclusions	All of the data acquired was utilized for analysis, and quality control filters for scRNA-seq and scATAC-seq data are specified in the Methods section.
Replication	We performed >10 independent biological replicates for each clinico-anatomical group (pediatric, adult, pontine, thalamic) via single cell profiling. We successfully replicated our findings across different tumors of each clinico-anatomical group.
Randomization	Acquisition of primary patient tumor samples was not randomized as all H3-K27M mutant diffuse midline gliomas across different age groups and anatomical locations were included in the study in an unbiased fashion.
Blinding	Blinding was not applicable as no effects of treatments or perturbations were assessed.

Reporting for specific materials, systems and methods

We require information from authors about some types of materials, experimental systems and methods used in many studies. Here, indicate whether each material, system or method listed is relevant to your study. If you are not sure if a list item applies to your research, read the appropriate section before selecting a response.

Materials & experimental systems

n/a	Involved in the study
☐	☒ Antibodies
☒	☐ Eukaryotic cell lines
☒	☐ Palaeontology and archaeology
☒	☐ Animals and other organisms
☐	☒ Human research participants
☒	☐ Clinical data
☒	☐ Dual use research of concern

Methods

n/a	Involved in the study
☒	☐ ChIP-seq
☐	☒ Flow cytometry
☒	☐ MRI-based neuroimaging

Antibodies

Antibodies used

Recombinant Anti-Histone H3 mutated K27M (Abcam ab190631, Lot: GR3333170-1, clone: EPR18340), dilution: 1 to 5000.
Goat Anti-Rabbit IgG H&L (Alexa Fluor 647) preadsorbed (Abcam ab150083, Lot: GR3370563-1), dilution: 1 to 4000.
Rabbit Anti-PDGFR alpha (Abcam ab234965, clone: EPR22059-270), dilution: 1 to 50.
Mouse Anti-NaBC1 (BCAS1) (Santa Cruz Biotechnology, sc-136342, clone: 5), dilution: 1 to 50.
Rat Anti-GFAP (Invitrogen, 13-0300, clone: 2.2B10), dilution: 1 to 50.
Mouse Anti-IBA1 (Thermo Fisher, clone: GT10312), dilution: 1 to 50.
Mouse Anti-CD63 (BioLegend, 353039, clone: H5C6), dilution: 1 to 50.
Mouse Anti-K67—Atto 550-RX047 (Akoya, #4250019, clone: B56), dilution: 1 to 200.
Rat Anti-CD44-BX005 - Atto 550-RX005 (Akoya, #4250002, clone: IM7), dilution: 1 to 50.

Validation

Anti-Histone H3 mutated K27M: Validated for Western Blotting, indirect ELISA, immunohistochemistry, immunofluorescence, immunoprecipitation and ChIP by the provider. Manufacturer references publications PMID: 31638150, PMID: 33239043, PMID: 31588023, and PMID: 29662203. IF protocol outlined in the methods section.
Goat Anti-Rabbit IgG H&L (Alexa Fluor 647) preadsorbed: Validated for immunohistochemistry, immunofluorescence, flow cytometry, and ELISA by the provider. Manufacturer references use in 49 publications (e.g. PMID: 33469673, PMID: 32616654) IF protocol outlined in the methods section.
Rabbit Anti-PDGFR alpha: validated for ELISA, Western Blot, immunohistochemistry, immunofluorescence, flow cytometry, immunoprecipitation by the provider. Referenced in PMID: 33805311.
Mouse Anti-NaBC1 (BCAS1): validated for Western Blot, immunofluorescence, and immunoprecipitation by the provider. Referenced in e.g. ,PMID: 31332391.
Rat Anti-GFAP: validated by provider for Western Blot, immunohistochemistry, immunocytochemistry, immunofluorescence, flow cytometry, immunoprecipitation, ELISA. Referenced in e.g., PMID: 27862351.
Mouse Anti-IBA1: validated by provider for Western Blot, immunohistochemistry, flow cytometry. Referenced in e.g., PMID: 34284798.
Mouse Anti-CD63: validated by provider for flow cytometry, immunohistochemistry. Referenced in e.g., PMID: 16410552.
Mouse Ki67-BX047 (B56)—Atto 550-RX047: validated for multiplexed IF (CODEX) in human and mouse tissues by the provider.
Rat Anti-CD44-BX005 - Atto 550-RX005 (Akoya, #4250002): validated for mutiplexed IF (CODEX) in human and mouse tissues by the provider.

Human research participants

Policy information about studies involving human research participants

Population characteristics

The covariate-relevant population and clinical characteristics of the human subjects whose data was used are available in Supplementary Table 1.

Recruitment

All primary patient glioma tissues were de-identified and obtained with properly informed consent of patients and/or their legal representatives treated at Boston Children's Hospital, Brigham and Women's Hospital, and collaborating institutions. Patients operated on for a glioma and/or their legal representatives were approached for participation and included in the study after confirmation of the H3-K27M mutation in an unbiased manner, thereby including all anatomical locations, age groups, clinical course, and treatments. Since clinical outcome was not assessed as an endpoint, we estimate any self-selection bias to be minimal.

Ethics oversight

This study was approved by the Institutional Review Board (IRB) at Boston Children's Hospital/Dana-Farber Cancer Institute (DFCI 10-417) and at affiliated research hospitals.

Note that full information on the approval of the study protocol must also be provided in the manuscript.

Flow Cytometry

Plots

Confirm that:

☒ The axis labels state the marker and fluorochrome used (e.g. CD4-FITC).

☒ The axis scales are clearly visible. Include numbers along axes only for bottom left plot of group (a 'group' is an analysis of identical markers).

☒ All plots are contour plots with outliers or pseudocolor plots.

☐ A numerical value for number of cells or percentage (with statistics) is provided.

Methodology

Sample preparation	Single-cell suspensions obtained from fresh tumors in PBS+1% BSA were stained with 0.5-1 µM calcein AM (Life Technologies, C3100MP) and 0.33 µM TO-PRO3 iodide (Life Technologies, T3605) for 15 min at RT and kept on ice. Single-cell sorting was performed on a SH800 (Sony) sorter using 488 nm (calcein AM, 530/30 emission filter) and 633 nm (TO-PRO-3, 665/30 emission filter) lasers. Viable cells were identified by positive staining for calcein AM and negative staining for TO-PRO-3. Doublets were discriminated based on back scatter area (BSC-A) versus back scatter width (BSC-W). Singlet viable cells were sorted into 96-well plates containing cold TCL buffer (Qiagen, 1031576), briefly spun down, snap frozen on dry ice, and stored at -80°C.<br>Single-nuclei suspensions extracted from frozen tumors were stained with 0.5 µM Vybrant DyeCycleTM Ruby Stain (Invitrogen, V10309) immediately before FACS. Intact nuclei were selected by positive staining for Ruby Stain on the SH800 sorter (633 nm laser, 665/30 nm emission filter). Doublets were excluded in the Ruby Stain area versus Ruby Stain height setting. Singlet nuclei were sorted into 96-well plates containing TCL buffer and 1% beta-mercaptoethanol, briefly spun down, snap frozen on dry ice and stored at -80 °C.
Instrument	SH800 (SONY) fluorescence-activated cell sorter with a 100 µm nozzle
Software	SONY SH800 software
Cell population abundance	Overall, we observed 10-60% viable cells (Calcein +, TO-PRO-3 -) following fresh tumor dissociation. For nuclei dissociated from frozen tumors, the Vybrant Ruby Stain positive event rate ranged from 10-90%.
Gating strategy	Live, single cells were identified by size (forward scatter), granularity (back scatter), singlet gating (back scatter area vs. back scatter width), positive staining for calcein and negative staining for TO-PRO-3.<br>Single nuclei were identified by positive staining for Vybrant Ruby Stain and singlet gating (Ruby Stain laser light area vs. Ruby Stain laser light height).

☒ Tick this box to confirm that a figure exemplifying the gating strategy is provided in the Supplementary Information.
